# Cancer Cell‐Intrinsic Cholesterol Induces Lipid‐Associated Macrophage Differentiation via SP1 Palmitoylation to Promote Prostate Cancer Progression

**DOI:** 10.1002/advs.202508588

**Published:** 2026-01-28

**Authors:** Shirong Peng, Weilong Lin, Zean Li, Ruilin Zhuang, Shengmeng Peng, Bingheng Li, Bingliang Chen, Yong Luo, Yuan Ou, Wei Zhuang, Tao Du, Kaiwen Li, Hai Huang

**Affiliations:** ^1^ Department of Urology Sun Yat‐sen Memorial Hospital Sun Yat‐sen University Guangzhou China; ^2^ Guangdong Provincial Key Laboratory of Malignant Tumor Epigenetics and Gene Regulation Guangdong‐Hong Kong Joint Laboratory for RNA Medicine Urology Department Sun Yat‐Sen Memorial Hospital Sun Yat‐Sen University Guangzhou China; ^3^ Department of Urology The Second Affiliated Hospital of Fujian Medical University Quanzhou Fujian China; ^4^ Department of Obstetrics and Gynecology Sun Yat‐Sen Memorial Hospital Sun Yat‐Sen University Guangzhou Guangdong China; ^5^ Guangdong Provincial Clinical Research Center for Urological Diseases Sun Yat‐Sen Memorial Hospital Sun Yat‐Sen University Guangzhou China; ^6^ Department of Urology The Sixth Affiliated Hospital of Guangzhou Medical University Qingyuan People's Hospital Qingyuan Guangdong China

**Keywords:** cholesterol, Lipid‐associated macrophages, midkine, S‐palmitoylation, Specificity protein 1

## Abstract

Cholesterol metabolism influences prostate cancer (PCa) progression, especially by affecting the tumor microenvironment. The present study demonstrated that cancer cell‐intrinsic cholesterol promoted the S‐palmitoylation of specificity protein 1 (SP1), enhancing SP1 nuclear translocation and driving the transcription and secretion of midkine (MDK), which in turn facilitated the differentiation of macrophages into a lipid‐associated phenotype. Furthermore, targeting cholesterol metabolism with simvastatin significantly reduced MDK levels, inhibited immunosuppressive macrophage polarization, and enhanced the efficacy of enzalutamide in vivo. These findings suggested that targeting the cancer cell‐intrinsic cholesterol‐induced immunosuppressive tumor microenvironment could be an effective strategy to improve therapeutic outcomes in prostate cancer patients.

## Graphical abstract

Cancer cell‐intrinsic cholesterol promotes the S‐palmitoylation of SP1, increasing its nuclear translocation and driving the transcription and secretion of MDK, which in turn facilitates the differentiation of macrophages into a lipid‐associated phenotype.

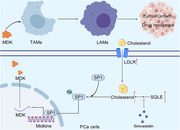

## 1 Introduction

The reprogramming of cholesterol metabolism plays a vital role in tumorigenesis. In addition to functioning as a component of cell membranes, cholesterol also participates in the synthesis of steroid hormones and other signaling molecules, thereby supporting the rapid proliferation and survival of cancer cells. Low‐density lipoprotein receptor (LDLR), high‐density lipoprotein receptor (HDLR), squalene epoxidase (SQLE), and HMG‐CoA reductase (HMGCR) are overexpressed in various tumors and are correlated with more aggressive tumor phenotypes, higher tumor stages, and an increased likelihood of metastasis [1, 2, 3]. In addition, elevated cholesterol levels within tumor tissues are closely associated with key oncogenic behaviors, such as drug resistance, metastasis, and malignant differentiation, in various malignancies, including hepatocellular carcinoma and breast cancer [4, 5, 6].

In the context of prostate cancer (PCa), alterations in cholesterol metabolism are frequent and have significant effects. Epidemiological studies have reported that men with elevated plasma cholesterol levels (≥ 240 mg/dL) have a markedly increased risk of developing high‐grade prostate cancer and are more susceptible to organ invasion [7, 8]. Furthermore, cholesterol‐lowering statins have demonstrated efficacy not only in reducing the incidence of prostate cancer but also in slowing disease progression and reducing prostate cancer‐specific mortality [9, 10]. These findings revealed that cholesterol is a modifiable risk factor for prostate cancer etiology and progression. The present study revealed significantly enhanced cholesterol metabolic activity in prostate cancer, characterized by increased synthesis and uptake of cholesterol in tumor cells, leading to cholesterol accumulation in cancer cells and progression of PCa, which is consistent with previous research [11, 12, 13]. However, further studies are needed to elucidate how accumulated cholesterol regulates the progression of prostate cancer.

S‐palmitoylation is the only reversible lipid modification, which involves palmitate attaching to cysteine residues through thioester bonds. The reversible nature of palmitoylation makes it a critical modulator of protein function, as it can influence various cellular processes by altering the interactions, transport and membrane associations of proteins [14, 15]. Aberrant palmitoylation can lead to dysregulated signaling, contributing to neoplasia and tumor progression. Various studies have shown that S‐palmitoylation modulates protein trafficking, subcellular localization, stability, and degradation by regulating conformational states and interaction networks. These molecular alterations subsequently perturb oncogenic signaling cascades, reprogram tumor metabolism, and reshape immune microenvironment dynamics, ultimately driving malignant transformation and therapeutic resistance in cancer systems [16, 17, 18, 19]. Moreover, S‐palmitoylation participates in the regulation of immune evasion mechanisms by modifying immune checkpoint proteins, such as PD‐L1, on cancer cells, thereby promoting an immunosuppressive tumor microenvironment [14, 20, 21].

The present study revealed significant accumulation of intracellular cholesterol during the progression of prostate cancer, facilitating palmitoylation of the SP1 transcription factor. The palmitoylation of SP1 triggered its translocation into the nucleus, increasing the transcription and secretion of MDK in tumor cells. Subsequently, MDK induced the differentiation of lipid‐associated macrophages, contributing to an immunosuppressive tumor microenvironment and promoting prostate cancer progression.

## 2 Results

### 2.1 Upregulation of Cholesterol Synthesis and Uptake in Cancer Cells Promotes PCa Progression

**FIGURE 1:**
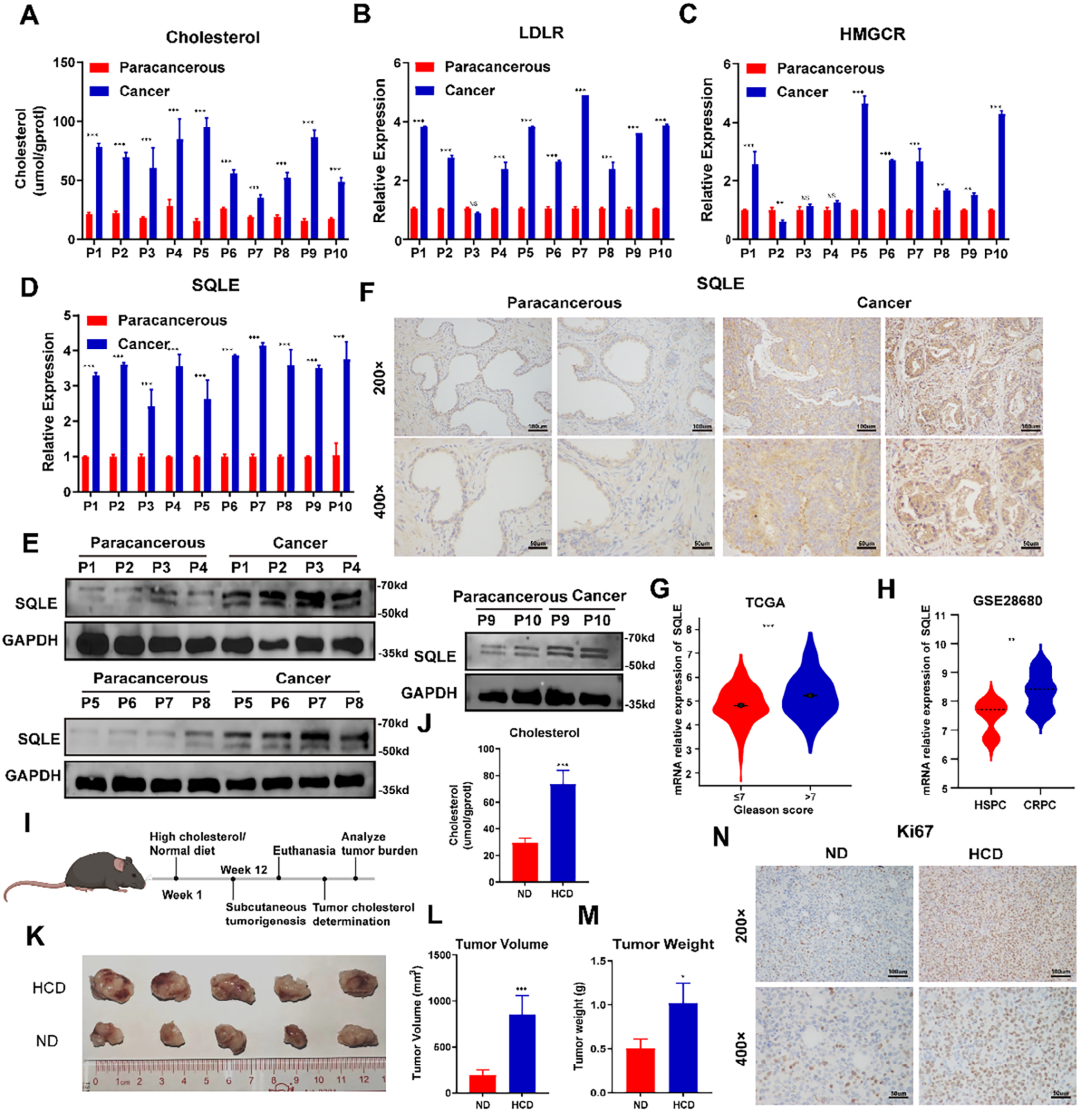
Altered cholesterol metabolism promotes PCa progression. (A) Cholesterol levels were significantly elevated in prostate cancer (PCa) tissues compared with normal prostate tissues. (B–D) Quantitative PCR analysis revealed increased mRNA expression levels of low‐density lipoprotein receptor (LDLR), 3‐hydroxy‐3‐methylglutaryl‐CoA reductase (HMGCR), and squalene monooxygenase (SQLE) in PCa tissues compared with normal prostate tissues. (E,F) Western blot (WB) and immunohistochemistry (IHC) analyses demonstrated upregulated SQLE protein expression in PCa. (G) Higher SQLE protein expression was associated with higher Gleason scores in PCa patients on the basis of data from TCGA. (H) Data from the GSE28680 dataset revealed upregulated SQLE expression in castration‐resistant prostate cancer (CRPC) compared with that in hormone‐sensitive prostate cancer (HSPC). (I) Schematic representation of an experimental model investigating the effects of a high‐cholesterol diet in mice. (J–N) High‐cholesterol diet (HCD) significantly promoted PCa growth in vivo (*n* = 5 per group). Data are presented as mean ± SD (*n* = 3), except for in vivo studies (*n* = 5). Statistical differences between the two groups were determined by Student's *t*‐test. ^*^ *p* < 0.05, ^**^ *p* < 0.01, ^***^ *p* < 0.001, and ns for non‐significant. Scale bars: 100 µm for 200X and 50 µm for 400X.

Alterations in cholesterol metabolism play crucial roles in the progression of prostate cancer. The inhibition of 3‐hydroxy‐3‐methylglutaryl‐CoA reductase (HMGCR), one of the rate‐limiting enzymes of cholesterol synthesis, inhibits the proliferation of PC cells. Similarly, squalene monooxygenase (SQLE), another rate‐limiting enzyme of cholesterol synthesis, also contributes to tumorigenesis, and its inhibition leads to reduced cancer growth, suggesting that therapeutic strategies targeting these enzymes may effectively suppress prostate cancer progression [22, 23]. To reveal the alterations in cholesterol metabolism in PCa, cholesterol content was measured in 10 paired prostate tumors and adjacesnt prostate tissues, which revealed markedly increased cholesterol levels in PCa tissues compared with matched adjacent prostate tissues (Figure 1A). To explore the details of increased cholesterol, we further analyzed the markers of cholesterol uptake (LDLR) and synthesis (HMGCR and SQLE) within tissues. Both cholesterol uptake and synthesis were upregulated in PCa, with the most significant upregulation observed in the SQLE cholesterol synthesis enzyme (Figure 1B–E). Interestingly, immunohistochemistry analysis further revealed that the increased expression of SQLE predominantly occurred in tumor epithelial cells. These findings suggested that cholesterol synthesis occurs mainly in tumor cells, highlighting the role of intracellular cholesterol in tumor cells in PCa (Figure 1F). Moreover, we analyzed data from TCGA database, which revealed a significantly greater expression of SQLE in PCa patients with higher Gleason scores (Figure 1G). Similar findings were observed when comparing castration‐resistant prostate cancer (CRPC) and hormone‐sensitive prostate cancer (HSPC) (Figure 1H). These results indicated that cholesterol accumulation is correlated with prostate cancer progression.

To determine the function of increased cholesterol in PCa progression, a normal diet (ND) or high‐cholesterol diet (HCD) was used to establish a mouse model (Figure 1I). Analysis of tumor tissues revealed that the HCD significantly elevated the cholesterol content in tumors (Figure 1J). Furthermore, the HCD markedly accelerated tumor growth in these mice, which indicated that elevated cholesterol levels promoted tumor progression (Figure 1K–N). Additionally, tumor cells overexpressing squalene monooxygenase (SQLE) were subcutaneously injected into mice to further examine the effects of altered cholesterol metabolism. These results were consistent with those of the HCD model, where SQLE overexpression significantly increased cholesterol levels within tumors and promoted tumor growth (Figure S1A–E). Taken together, these findings revealed increased synthesis and uptake of cholesterol in tumor cells during the progression of prostate cancer, leading to an increase in cholesterol levels and further promotion of tumor growth.

### 2.2 Cancer Cell‐Intrinsic Cholesterol Promotes the Differentiation of Lipid‐Associated Macrophages in PCa

**FIGURE 2:**
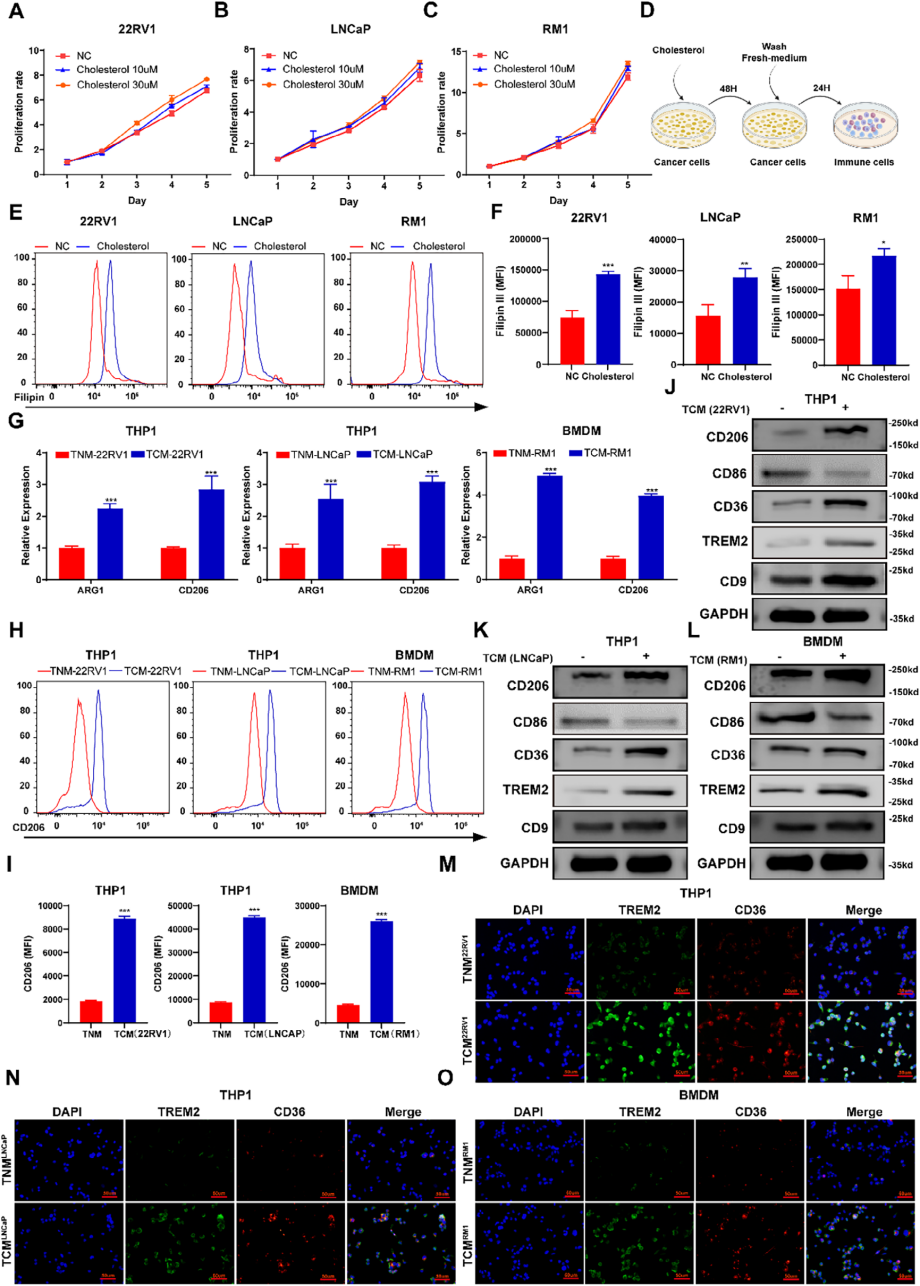
Lipid‐associated macrophage differentiation is induced by PCa tumor cells with high cholesterol levels. (A–C) Cholesterol did not significantly promote the growth of PCa cells in vitro. (D) Schematic illustration of the indirect coculture model. (E,F) Cholesterol pretreatment increased the cholesterol level in PCa cells. (G) Quantitative PCR analysis demonstrated that conditioned medium from cholesterol‐pretreated PCa cells increased the mRNA levels of ARG1 and CD206 in macrophages. (H,I) Flow cytometry analysis revealed upregulated CD206 expression on macrophages following exposure to conditioned medium from cholesterol‐pretreated PCa cells. (J–L) WB revealed that the conditioned medium of cholesterol‐pretreated tumor cells increased the expression levels of CD206, CD36, TREM2 and CD9 in macrophages but reduced the expression levels of CD86. (M–O) Immunofluorescence staining revealed elevated expression levels of CD36 and TREM2 in macrophages treated with conditioned medium from cholesterol‐pretreated PCa cells. Scale bar: 50 µm. Data are presented as mean ± SD (*n* = 3). Statistical analyses were performed by comparing with controls using Student's *t*‐test. ^*^ *p* < 0.05, ^**^ *p* < 0.01, and ^***^ *p* < 0.001.

As demonstrated above, we observed that enhanced cholesterol biosynthesis and uptake elevated intracellular cholesterol levels and promoted tumor progression in vivo. Consequently, we sought to determine whether these factors directly influence tumor cell growth in vitro. Notably, overexpression of LDLR did not significantly enhance prostate cancer cell proliferation (Figure S2A–C). This finding aligns with a previous report in breast cancer, in which LDLR silencing attenuated tumor growth in mouse models but did not affect cell proliferation under standard culture conditions [1]. Furthermore, while SQLE overexpression resulted in a modest increase in prostate cancer cell growth in vitro (Figure S2E–G), this effect was considerably less pronounced than that observed in vivo. To rule out the possibility that the in vitro results were confounded by cholesterol‐independent effects of the overexpression constructs, we directly treated tumor cells with exogenous cholesterol to elevate intracellular cholesterol levels. Consistent with our previous results, increased intracellular cholesterol levels did not significantly promote tumor growth (Figure 2A–C). These findings implied that while cholesterol may play a role in tumor development, its accumulation may not directly promote tumor growth. One of the primary factors contributing to the differences in experimental outcomes between in vivo and in vitro experiments is the tumor microenvironment [24]. To further investigate the potential effects of cancer cell‐intrinsic cholesterol on immune cells, we established an indirect coculture model (Figure 2D). Tumor cells were first treated with exogenous cholesterol for 48 h to increase the intracellular cholesterol level (Figure 2D–F). The PCa cells were then thoroughly washed and cultured with fresh medium to remove residual cholesterol. After an additional 24 h of culture, the conditioned medium was collected, and the cholesterol content in the conditioned medium was measured. Although cholesterol pretreatment increased the cholesterol content inside the cells, it did not significantly change the cholesterol content in the conditioned medium (Figure S2H–J). The conditioned medium was then used to stimulate macrophages and T cells, the key immune cells involved in PCa. The conditioned medium of cholesterol‐pretreated cells promoted the polarization of macrophages toward an immunosuppressive phenotype but had no significant effect on the cytotoxic activity of T cells (Figure 2G–I and Figure S2K–M). Immunosuppressive macrophages activate the β‐catenin/STAT3 signaling pathway, secrete cytokines, and create a tumor‐supportive microenvironment, promoting tumor progression [25, 26]. Lipid‐associated macrophages (LAMs) are a group of immunosuppressive cells that play a critical role in the tumor microenvironment, particularly within tumors undergoing metabolic reprogramming [6, 27]. However, the precise mechanisms by which macrophages within tumor tissues differentiate into LAMs remain unclear. Given that significant alterations in cholesterol metabolism occurred within prostate cancer cells, we evaluated whether the metabolic changes in tumor cells act as initiating drivers of LAMs differentiation. We performed WB and immunofluorescence to detect the phenotype of macrophages treated with conditioned medium from cholesterol‐pretreated cells. The conditioned medium increased the expression of CD206, TREM2, CD36 and CD9 but decreased the expression of CD86 in macrophages, leading to LAMs differentiation (Figure 2J–O). Although we observed no significant differences in cholesterol content within the collected conditioned media, we sought to further investigate whether cholesterol directly promotes the differentiation of LAMs. Upon directly treating macrophages with cholesterol and examining relevant markers, we found that cholesterol treatment alone did not induce LAM differentiation. Thus, these results indicated that cancer cell‐intrinsic cholesterol promotes lipid‐associated macrophage differentiation indirectly to promote PCa progression.

### 2.3 Cancer Cell‐Intrinsic Cholesterol Induces MDK Secretion to Increase Lipid‐Associated Macrophage Differentiation in PCa

**FIGURE 3:**
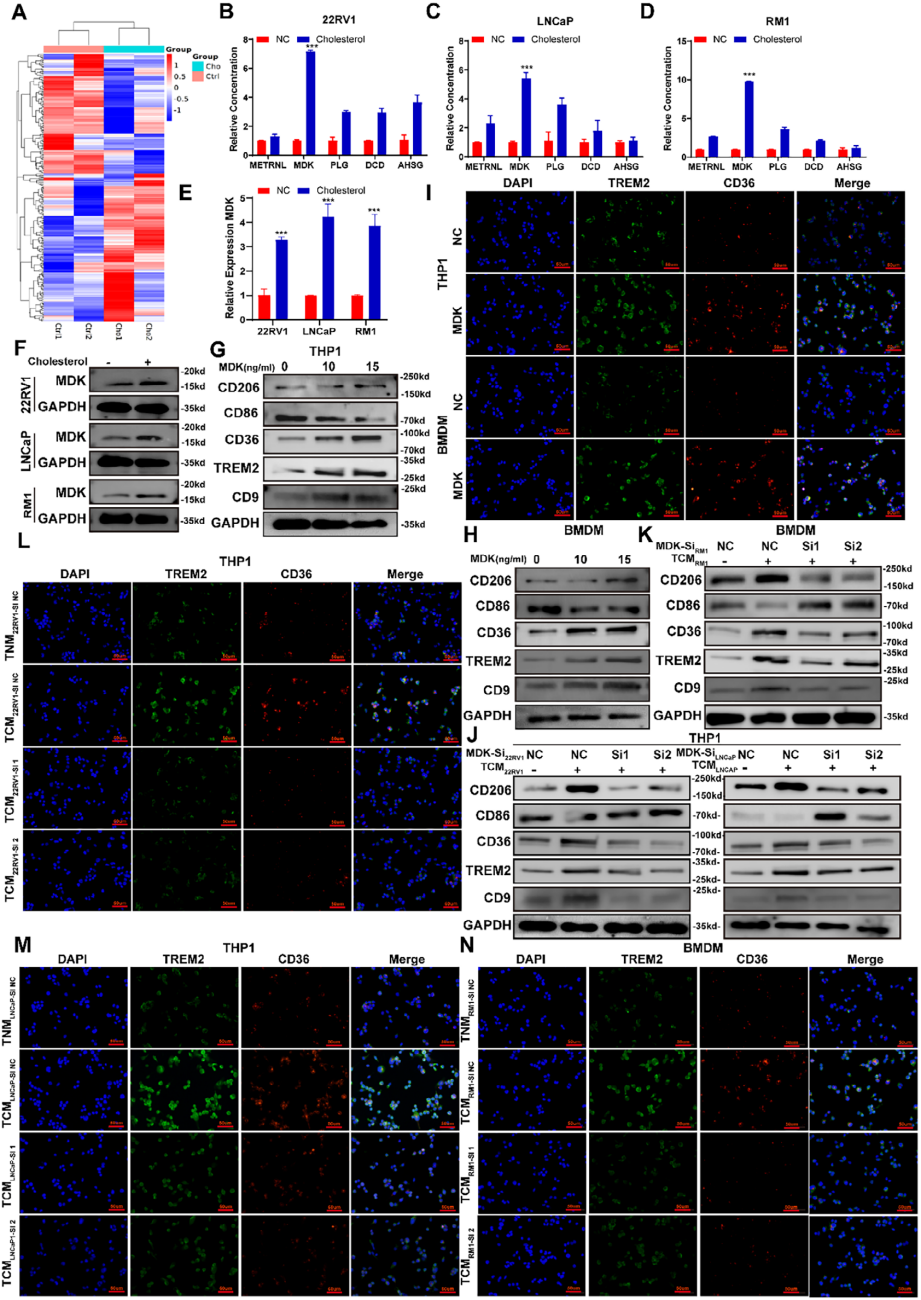
Cholesterol promotes the secretion of MDK in PCa cells, leading to the differentiation of lipid‐associated macrophages. (A) Heatmap of the protein profiles of the prostate cancer (PCa) cell supernatants. (B–D) ELISA quantification of plasminogen (PLG), dermcidin (DCD), meteorin‐like protein (METRNL), α2‐HS glycoprotein (AHSG), and midkine (MDK) levels in the supernatants of PCa cells pretreated with or without cholesterol. (E,F) The expression of MDK in PCa cells pretreated with or without cholesterol was detected via PCR and WB analyses. (G,H) WB analysis of CD206, CD36, TREM2, CD9, and CD86 expression in THP‐1 cells or BMDMs treated with or without recombinant MDK protein. (I) Immunofluorescence staining of CD36 and TREM2 expression in THP‐1 cells or BMDMs treated with or without recombinant MDK protein. (J–N) PCa cells with or without MDK knockdown were pretreated with or without cholesterol, and the conditioned medium was subsequently collected to stimulate macrophages. The expression levels of CD206, CD36, TREM2, CD9, and CD86 in macrophages were analyzed by WB (J,K) and immunofluorescence (L–O) analyses. Scale bar: 50 µm. Data are presented as mean ± SD (*n* = 3). Statistical differences between the two groups were determined by Student's *t*‐test. ^*^ *p* <0.05, ^**^ *p* < 0.01, and ^***^ *p* < 0.001.

In the tumor microenvironment, fibroblasts, immune cells, and tumor cells regulate tumor progression by secreting growth factors, chemokines, and cytokines. To identify the specific mediators by which cholesterol promotes the secretion of PCa cells, quantitative proteomic analysis was conducted. Cholesterol‐treated PCa cells presented increased secretion of several cytokines, including plasminogen (PLG), dermcidin (DCD), meteorin‐like protein (METRNL), α2‐HS glycoprotein (AHSG), and midkine (MDK). Among these cytokines, MDK exhibited the most significant upregulation (Figure 3A–D). Further analysis demonstrated that cholesterol treatment also increased the expression of MDK in PCa cells at both the mRNA and protein levels (Figure 3E,F). Next, we analyzed the relationships of the expression of PLG, DCD, METRNL, AHSG, and MDK with the prognosis of PCa patients and found that higher expression of MDK was closely related to poorer prognosis in PCa patients (Figure S3A–F). Midkine (MDK), a soluble heparin‐binding growth factor, is highly elevated in various malignancies and is known to drive inflammatory responses and tumor progression. To further investigate the clinical relevance of MDK in prostate cancer, we analyzed a cohort from Sun Yat‐sen Memorial Hospital. We observed that elevated MDK expression was significantly correlated with higher Gleason scores (Figure S3G,H). Given this association, we hypothesized that MDK might serve as the primary mediator by which cholesterol drives the differentiation of LAMs. To validate this hypothesis, we treated macrophages with recombinant MDK protein, which significantly promoted LAMs differentiation (Figure 3G–I). Furthermore, silencing MDK in prostate cancer cells using siRNA significantly abrogated the LAM differentiation induced by conditioned medium from cholesterol‐pretreated cells (Figure 3J–N). In addition, we evaluated the efficacy of an MDK inhibitor (iMDK) in mouse models. We found that iMDK significantly inhibited LAMs differentiation and attenuated prostate tumor growth, while also enhancing the sensitivity of tumors to the androgen receptor inhibitor enzalutamide (Figure S3I–L). Collectively, these findings indicate that intracellular cholesterol accumulation in cancer cells induces MDK secretion, thereby triggering lipid‐associated macrophage differentiation and facilitating PCa progression.

### 2.4 Cancer Cell‐Intrinsic Cholesterol Promotes MDK Synthesis and Secretion in PCa Cells by Enhancing the Nuclear Translocation of SP1

**FIGURE 4:**
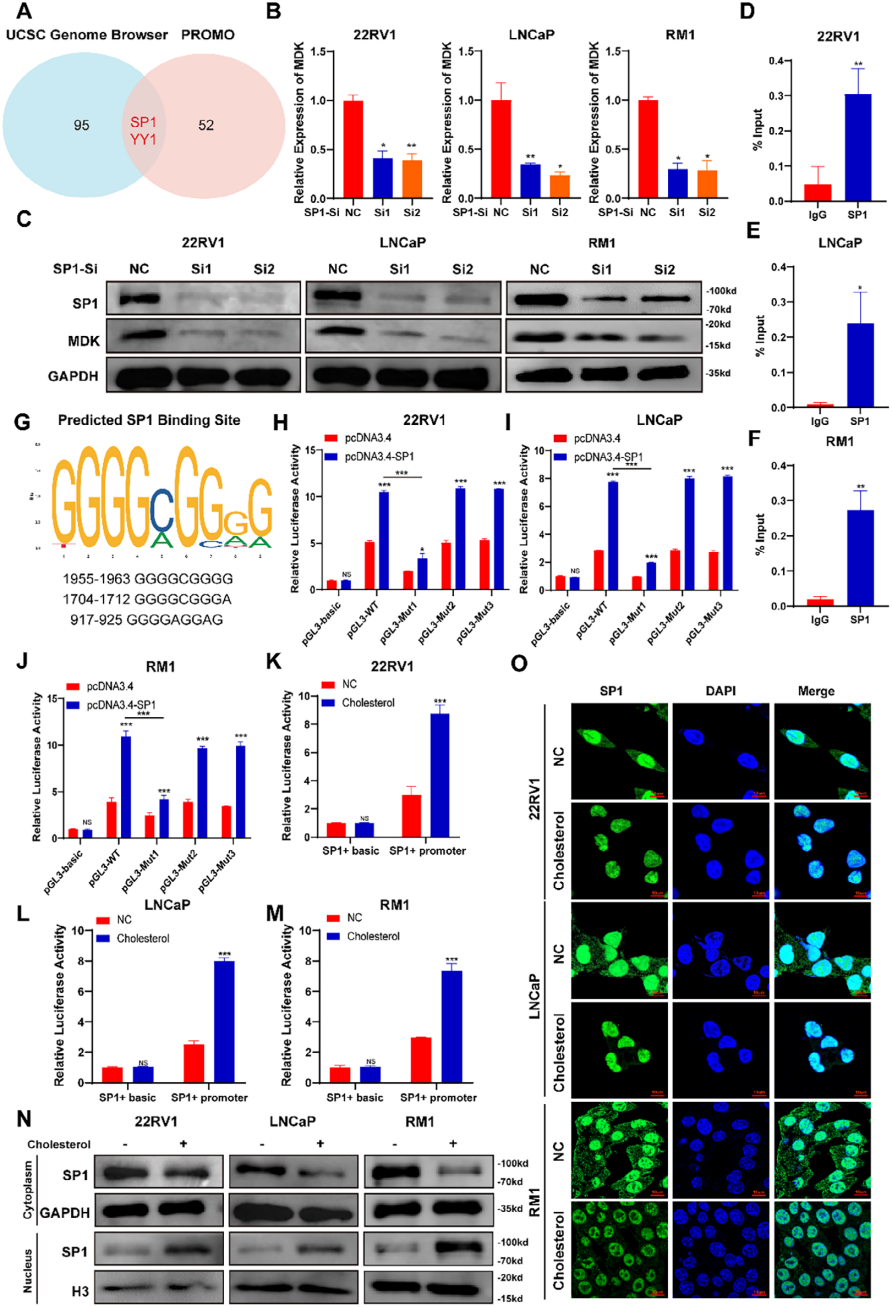
Cancer cell‐intrinsic cholesterol enhances the nuclear translocation of SP1, promoting the transcription of MDK. (A) Venn diagram illustrating the screened transcription factors involved in midkine (MDK) regulation, identifying SP1 and YY1 as potential regulators in prostate cancer (PCa) cells. (B,C) SP1 knockout reduced MDK expression at both the mRNA and protein levels. (D–F) ChIP experiments confirmed the binding of SP1 to the MDK promoter. G Prediction of potential SP1‐binding sites within the MDK promoter region in Homo sapiens, as identified through the JASPAR database. (H–J) Dual‐luciferase reporter assays were conducted to assess SP1 binding to the MDK promoter following mutation of various binding sites. (K–M) Dual‐luciferase reporter assays further confirmed that cholesterol enhanced the binding between SP1 and the MDK promoter. (N,O) Nuclear and cytoplasmic protein extraction, along with immunofluorescence analysis, demonstrated that cholesterol promoted SP1 nuclear translocation. Scale bar: 10 µm. Data are presented as mean ± SD (*n* = 3). Statistical differences between two groups were determined by Student's *t*‐test (B, siNC as control), whereas comparisons across multiple groups were assessed via one‐way ANOVA with Dunnett's post hoc test for multiple comparisons (H‐M). ^*^ *p* < 0.05, ^**^ *p* < 0.01, ^***^ *p* < 0.001, and ns for non‐significant.

Given that cholesterol increased MDK mRNA levels in PCa cells (Figure 3E), we screened the UCSC Genome Browser and PROMO databases to understand how cholesterol regulates MDK transcription. Both SP1 and YY1 were identified as potential transcription factors that regulate MDK transcription in PCa cells (Figure 4A). We then found that SP1 knockdown, rather than YY1 knockdown, significantly reduced the expression of MDK in tumor cells (Figure 4B,C and Figure S4A–C). Additionally, ELISA analysis corroborated that silencing SP1 significantly attenuated MDK secretion by prostate cancer cells (Figure S4D). SP1, a Cys2His2‐zinc finger protein (C2H2‐ZNF) transcription factor, is crucial for cell differentiation, apoptosis, and carcinogenesis [28, 29]. Subsequent chromatin immunoprecipitation (ChIP) experiments confirmed that SP1 binds to the MDK promoter and directly regulates its transcription (Figure 4D–F). Thus, these findings confirmed that SP1 is the key regulator of MDK transcription in PCa cells.

Three potential SP1‐binding sites were identified in the MDK promoter through the JASPAR database (Figure 4G; Figure S4E). To pinpoint the major binding site, the identified binding sites within the MDK promoter were mutated, and dual‐luciferase reporter assays were conducted to detect the binding between SP1 and the MDK promoter. Mutation 1 significantly reduced the binding of SP1 to the MDK promoter (Figure 4H–J). These findings suggested that SP1 regulates MDK transcription mainly by recognizing and binding to the GGGGCGGGG sequence in the MDK promoter of Homo sapiens and the CCCCTCCCCC sequence in the MDK promoter of Mus musculus. Furthermore, dual‐luciferase reporter assays demonstrated that cholesterol enhanced the binding between SP1 and the MDK promoter (Figure 4K–M). To further investigate how cholesterol promotes the binding of SP1 to the MDK promoter, we examined the levels and localization of SP1 in cholesterol‐pretreated cells. The expression of SP1 was not affected by cholesterol (Figure S4F), but the distribution of SP1 within the nucleus was significantly increased by cholesterol (Figure 4N–O). These results suggested that cancer cell‐intrinsic cholesterol enhances the nuclear distribution of SP1, which facilitates the binding of SP1 to the MDK promoter, thereby promoting MDK transcription.

### 2.5 Cholesterol Promotes SP1 Nuclear Translocation via S‐palmitoylation to Enhance MDK Synthesis and Secretion

**FIGURE 5:**
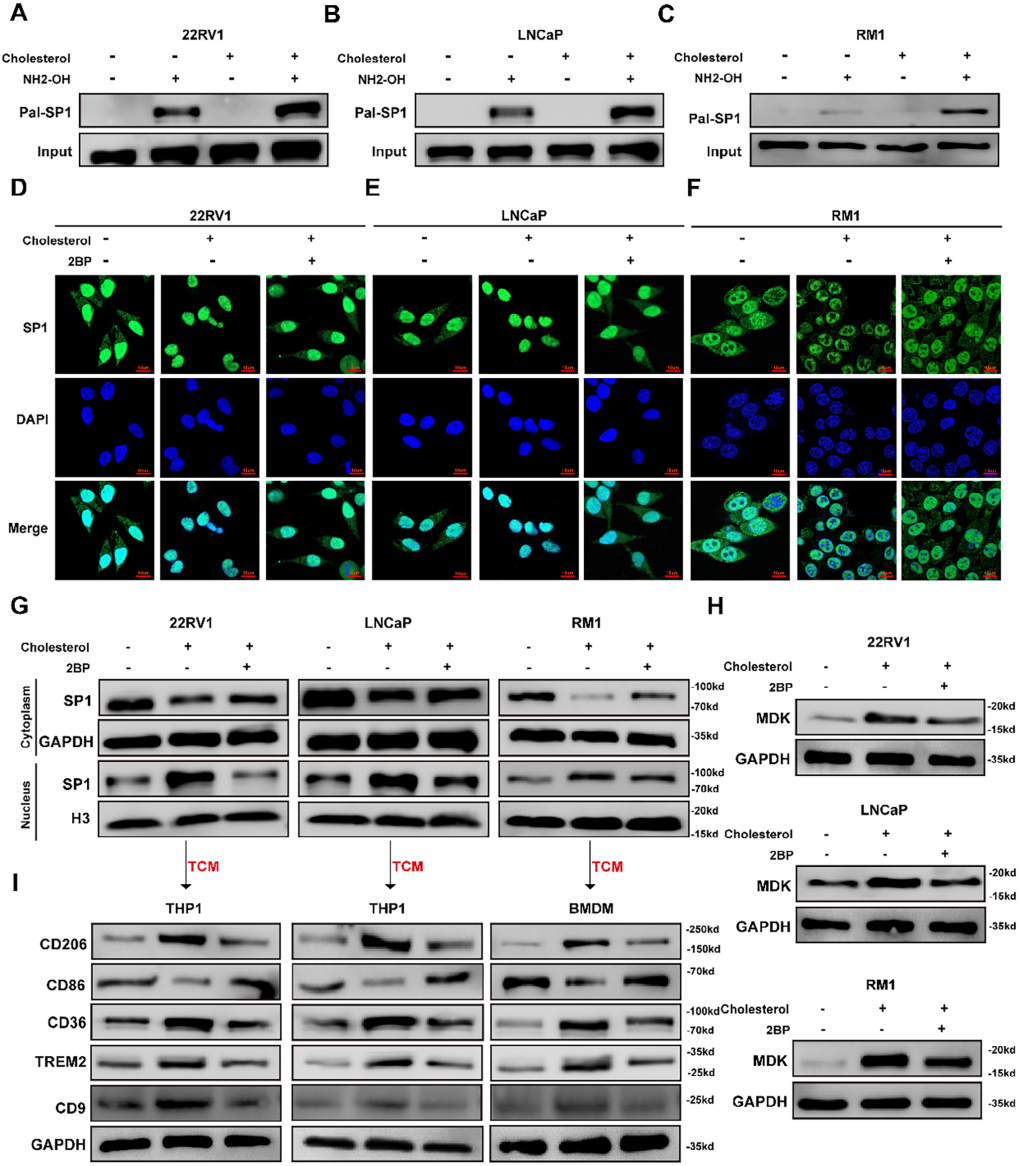
Cholesterol promotes SP1 nuclear translocation via S‐palmitoylation. (A–C) Acyl‐biotin exchange (ABE) assays demonstrated that cholesterol promoted the S‐palmitoylation of SP1. D‐G Nuclear and cytoplasmic protein extraction (G) combined with immunofluorescence (D–F) analysis indicated that 2‐bromopalmitate (2BP) reversed the cholesterol‐induced increase in SP1 nuclear translocation. Scale bar: 10 µm. (H) WB analysis revealed that 2BP treatment counteracted the cholesterol‐mediated increase in MDK expression. (I) WB analysis revealed that 2BP treatment of prostate cancer (PCa) cells reversed the differentiation of lipid‐associated macrophages, which was promoted by conditioned medium from cholesterol‐pretreated PCa cells.

S‐palmitoylation, the only reversible lipid modification of proteins, plays a dynamic role in regulating protein trafficking, localization, and the formation of protein complexes [14, 15, 30]. Cholesterol is one of the key lipids within cells [31, 32], and changes in cholesterol metabolism may cause a shift in the intracellular lipid balance. Moreover, a study has shown that the binding of cholesterol to FZD5 is necessary for its palmitoylation [33], suggesting that cholesterol may be related to the palmitoylation of proteins. Thus, to determine whether cholesterol promotes SP1 nuclear translocation via S‐palmitoylation, we conducted an acyl‐biotin exchange (ABE) assay, which demonstrated that cholesterol increased the palmitoylation of SP1 (Figure 5A–C). Moreover, immunofluorescence staining revealed that 2‐bromopalmitate (2BP), a palmitoylation inhibitor that targets DHHC (Asp‐His‐His‐Cys) protein palmitoyltransferase, effectively reversed the cholesterol‐induced nuclear translocation of SP1 (Figure 5D–F). Consistent results were obtained through nuclear and cytoplasmic protein extraction analysis (Figure 5G). These findings indicated that S‐palmitoylation plays a pivotal role in cholesterol‐mediated regulation of SP1 nuclear translocation. As expected, 2BP attenuated the cholesterol‐induced increase in MDK synthesis and secretion within tumor cells (Figure 5H; Figure S5A), resulting in a diminished ability of the conditioned medium from cholesterol‐pretreated cells to promote the differentiation of lipid‐associated macrophages (Figure 5I; Figure S5B–D). Collectively, these results demonstrated that cholesterol enhances SP1 palmitoylation, promoting its nuclear translocation, which in turn drives MDK synthesis and the differentiation of lipid‐associated macrophages.

### 2.6 Cholesterol Palmitoylates SP1 at Cysteine 691 to Promotes its Nuclear Translocation

**FIGURE 6:**
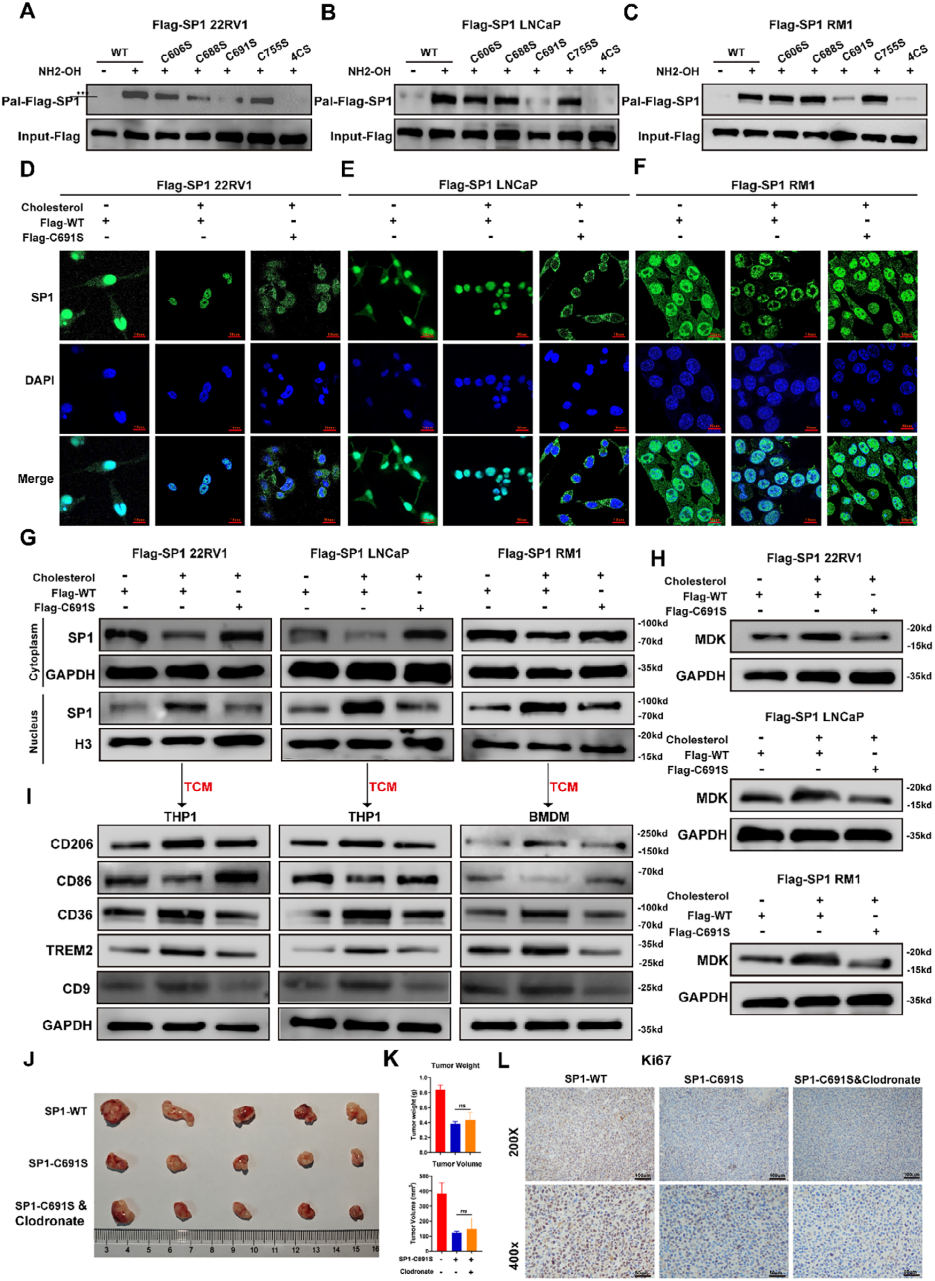
Cholesterol palmitoylates SP1 at cysteine 691 to promote the nuclear translocation of SP1. (A–C) Constructs with Flag‐tagged WT or SP1 with Cys606, Cys688, Cys691, or Cys755 mutations were transfected into PCa cells, and ABE assays demonstrated that the Cys691S mutation significantly reduced SP1 palmitoylation. (D–G) Nuclear and cytoplasmic protein extraction, along with immunofluorescence analysis, revealed that the Cys691S mutation reversed the cholesterol‐induced nuclear translocation of SP1 in PCa cells. Scale bar: 10 µm. (H) WB analysis revealed that the Cys691S mutation counteracted the cholesterol‐induced increase in MDK expression. (I) WB analysis demonstrated that the Cys691S mutation in PCa cells reversed the differentiation of lipid‐associated macrophages, which was induced by conditioned medium from cholesterol‐pretreated PCa cells. (J–L) Cys691S mutation inhibited the growth of PCa in vivo via macrophages (Clodronate: Clodronate Liposomes, *n* = 5 per group). Data are presented as mean ± SD (*n* = 5). Statistical differences between multiple groups were assessed via one‐way ANOVA with Dunnett's post hoc test for multiple comparisons. ^*^ *p* < 0.05, ^**^ *p* < 0.01, ^***^ *p* < 0.001, and ns for non‐significant.

As mentioned above, Cholesterol enhanced SP1 palmitoylation, we then investigated the palmitoylation sites within SP1. As the SP1 protein sequences of humans and mice are highly homologous, we used the human SP1 protein sequence for further research. Four potential sites—Cys606, Cys688, Cys691 and Cys755—were identified via GPS‐Palm and CSS‐Palm 4.0, and these cysteine residues were selected for further investigation as potential palmitoylation sites involved in regulating SP1 activity and localization. To reveal the main palmitoylation sites of SP1, four cysteine residues were individually or simultaneously mutated to serine residues in SP1. Assessment of palmitoylated SP1 revealed that the C691S mutation, along with the mutation of all four cysteine residues, nearly completely abolished SP1 palmitoylation (Figure 6A–C), indicating that Cys691 is the major site responsible for SP1 palmitoylation. Next, we investigated whether palmitoylation at the C691 site is essential for cholesterol to promote SP1 nuclear translocation. Both immunofluorescence staining and nuclear‐cytoplasmic protein extraction revealed that the C691S mutation reversed the cholesterol‐induced nuclear translocation of SP1 (Figure 6D–F). Additionally, compared with wild‐type SP1, the C691S mutation attenuated the cholesterol‐induced increase in MDK synthesis and secretion in tumor cells and diminished the ability of conditioned medium from cholesterol‐pretreated cells to promote the differentiation of lipid‐associated macrophages (Figure 6G–I; Figure S6A–D). Moreover, the C691S mutation inhibited tumor growth exclusively in vivo, with no significant effect observed in vitro (Figure S6E). Notably, macrophage depletion via clodronate liposomes (Figure S6F,G) did not further potentiate the growth inhibition caused by the C691S mutation (Figure 6J–L), suggesting that the mutation exerts its effects through the same macrophage‐dependent axis. Collectively, these results suggest that palmitoylation at Cys691 is critical for the cholesterol‐mediated regulation of SP1 activity and its downstream modulation of tumor‐immune interactions.

### 2.7 Targeting Cholesterol Metabolism Remodels Lipid‐Associated Macrophages Differentiation to Enhance the Efficacy of Enzalutamide in PCa

**FIGURE 7:**
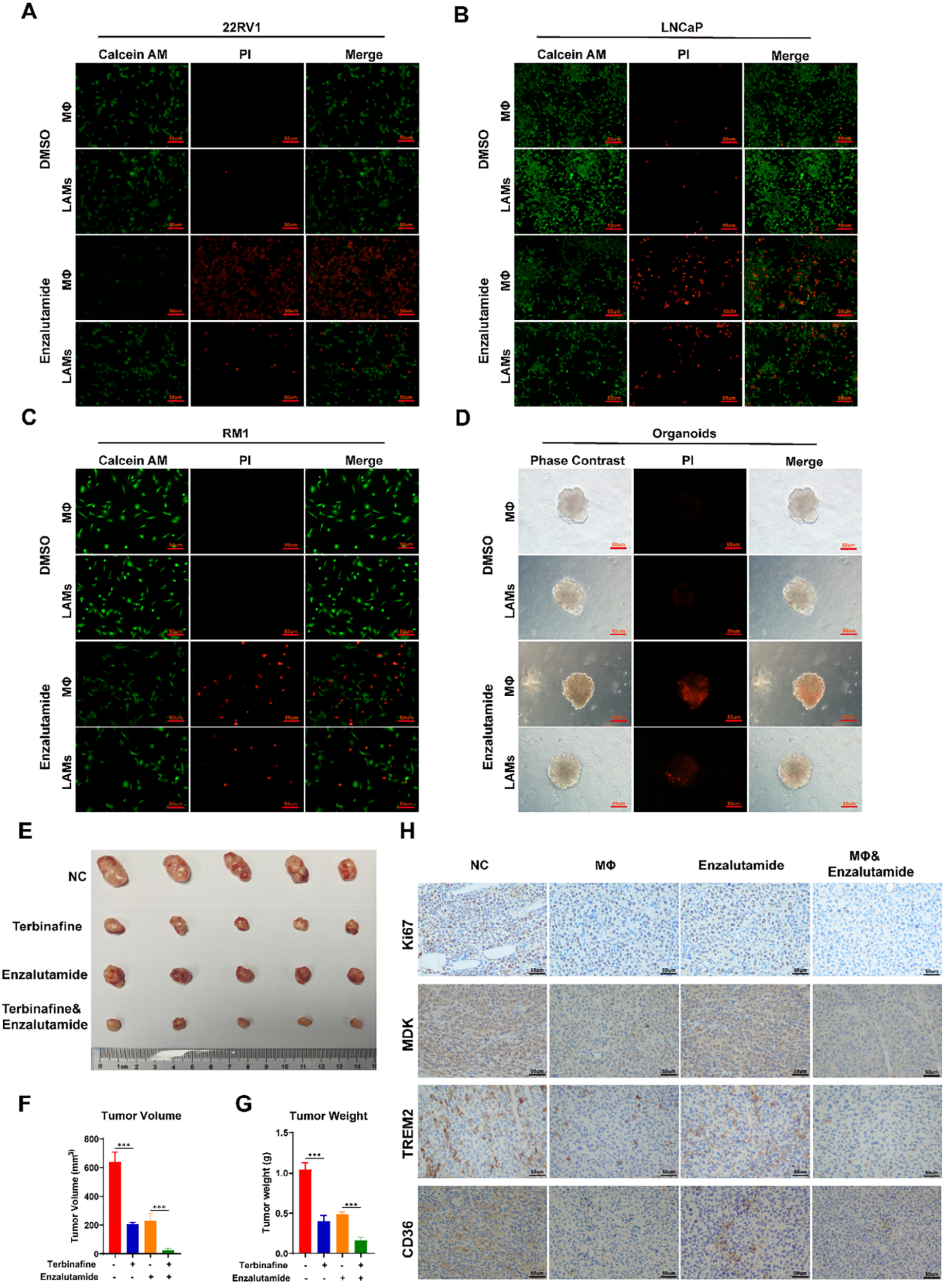
Targeting cholesterol metabolism enhances the efficacy of enzalutamide in PCa. (A–C) Prostate cancer (PCa) cells were co‐cultured with lipid‐associated macrophages induced by conditioned medium from cholesterol‐pretreated PCa cells, followed by treatment with enzalutamide. Calcein AM/PI staining indicated that lipid‐associated macrophages diminished the therapeutic efficacy of enzalutamide in PCa cells. (D) PCa organoids were co‐cultured with lipid‐associated macrophages, and PI staining revealed that lipid‐associated macrophages reduced the therapeutic effect of enzalutamide on tumor organoids. (E–H) Targeting cholesterol metabolism in cancer cells using simvastatin enhanced the efficacy of enzalutamide treatment in vivo (*n* = 5 per group). Data are presented as mean ± SD (*n* = 5). Statistical differences between multiple groups were assessed via one‐way ANOVA with Dunnett's post hoc test for multiple comparisons. Scale bar: 50 µm. ^*^ *p* < 0.05, ^**^ *p* < 0.01, and ^***^ *p* < 0.001.

Tumor‐associated macrophages (TAMs) play a critical role in the development of drug resistance across various tumors. These macrophages mediate resistance to chemotherapy and radiotherapy by secreting cytokines and activating antiapoptotic pathways, thereby increasing the survival and resistance of tumor cells to therapeutic interventions [34, 35, 36]. A recent study has revealed that immunosuppressive tumor‐associated macrophages are associated with enzalutamide resistance in prostate cancer [37]. Given that SQLE expression was elevated in CRPC (Figure 1H), we further investigated whether tumor cell‐induced lipid‐associated macrophages contribute to androgen receptor pathway inhibitor (ARPI) therapy resistance in PCa. As we could see, cancer cell‐intrinsic cholesterol‐induced lipid‐associated macrophages reduced enzalutamide‐induced prostate cancer cell death within the coculture system (Figure 7A–C). These findings suggested that the presence of LAMs contributed to therapeutic resistance by enhancing the survival of prostate cancer cells, underscoring the potential of targeting cholesterol metabolism to improve the efficacy of enzalutamide treatment. Consistent results were observed in prostate cancer organoid models (Figure 7D). Furthermore, we co‐injected tumor cells with either naive macrophages or induced LAMs into mice to evaluate the therapeutic efficacy of enzalutamide. The results demonstrated that LAMs promoted tumor growth and significantly attenuated the tumor response to enzalutamide (Figure S7A–C). To delineate the impact of cholesterol regulation on resistance to ARPI therapy, we utilized the specific SQLE inhibitor terbinafine in mouse models. Terbinafine treatment significantly impaired the differentiation of lipid‐associated macrophages (LAMs), attenuated tumor growth, and sensitized tumors to enzalutamide (Figure 7E–H).

Simvastatin is a clinically proven, safe, and effective cholesterol‐lowering agent. Although its primary target is not SQLE, its established safety profile facilitates rapid clinical translation. We therefore performed similar in vivo assays using simvastatin and observed that it similarly inhibited LAM differentiation and enhanced the efficacy of enzalutamide (Figure S7E–H). This effect may be attributable to the simvastatin‐mediated reduction in systemic cholesterol synthesis, which subsequently decreases cholesterol uptake by tumor cells and blocks the associated downstream signaling cascades. Collectively, our findings suggest that targeting cholesterol metabolism offers a viable approach to improve the therapeutic outcome of ARPI therapy in prostate cancer.

## 3 Discussion

**FIGURE 8:**
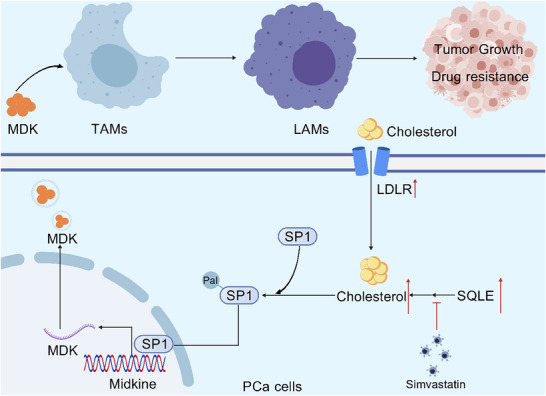
Cancer cell‐intrinsic cholesterol induces lipid‐associated macrophage differentiation via SP1 palmitoylation to promote prostate cancer progression.

The critical role of cholesterol metabolism in prostate cancer (PCa) has been previously reported. However, the specific mechanisms by which alterations in cholesterol metabolism promote PCa progression remain unclear. Further investigations are needed to elucidate how these metabolic changes contribute to tumor development and therapeutic resistance. The present study demonstrated that the upregulation of cholesterol synthesis and uptake in PCa cells enhances cholesterol accumulation, which in turn promotes the S‐palmitoylation of SP1, facilitating its nuclear translocation and subsequent regulation of midkine (MDK) synthesis, driving the polarization of macrophages toward a lipid‐associated phenotype (Figure 8).

Lipid‐associated macrophages, a group of immunosuppressive macrophages, are characterized by high expression of CD206, TREM2, and CD36 but low expression of CD86. These markers distinguish a subset of macrophages involved in promoting tumor progression and creating an immunosuppressive tumor microenvironment. Recent research has suggested that MDK derived from tumor cells may promote the differentiation of lipid‐associated macrophages [38, 39]. A previous study on NSCLC leptomeningeal metastasis demonstrated that tumor‐derived MDK promotes M2 macrophage polarization, enhancing both lipid metabolism and phagocytic function [40]. Furthermore, that study revealed that such macrophages mediate therapeutic resistance through the modulation of T cells and intercellular communication, which offers a plausible explanation for the reduced enzalutamide sensitivity mediated by LAMs in our findings. Given the extensive body of literature already confirming the diverse mechanisms by which LAMs facilitate drug resistance, we did not further characterize the specific downstream resistance pathways in this study. However, the upstream factors regulating MDK secretion by tumor cells have remained elusive in previous reports. Elucidating this regulatory mechanism represents one of the primary contributions of our current work. The present study revealed a novel regulatory mechanism for MDK secretion in which intracellular cholesterol promotes the nuclear translocation of the SP1 transcription factor via palmitoylation, thereby enhancing the transcriptional synthesis of MDK. Furthermore, we identified the binding site between SP1 and the MDK promoter, providing new insights into this regulatory pathway.

Many studies have suggested that changes in the intracellular localization of proteins are dependent on posttranslational modifications. Reversible protein modifications occur faster than the synthesis of new proteins, enabling cells or organisms to quickly meet the needs of different physiological processes. As a result, protein activity, stability, and intracellular localization are often regulated by reversible posttranslational modifications, such as phosphorylation/dephosphorylation, acetylation/deacetylation, and ubiquitination/deubiquitination, among others [41, 42]. S‐Palmitoylation, a reversible lipid modification, has gained increasing attention from researchers in recent years. An expanding number of proteins have been identified as substrates for palmitoylation, and their functional roles are gradually being elucidated. One of the key findings from the present study was the identification of Cys691 as the primary site for SP1 palmitoylation, which is essential for its cholesterol‐induced nuclear translocation. The present results demonstrated that mutations at this site significantly impair the cholesterol‐mediated regulation of SP1 activity, reducing MDK synthesis and the differentiation of lipid‐associated macrophages. These data provide new insights into how cholesterol metabolism influences transcriptional regulation and immune evasion in PCa via S‐palmitoylation. Unfortunately, we have not yet identified the specific mechanism by which cholesterol promotes SP1 palmitoylation, which will be one of the main focuses of our future research. However, when cholesterol derivatives combine with liver X receptors (LXRs), they activate SREBF1 and SCD1 to upregulate fatty acid synthesis [43]. Accumulated cholesterol may promote SREBF1‐ or SCD1‐mediated palmitic acid synthesis to increase S‐palmitoylation.

Our findings demonstrate that targeting MDK or cholesterol metabolism effectively inhibits immunosuppressive and lipid‐associated macrophage differentiation, and enhances the therapeutic efficacy in PCa. To address the clinical challenge of endocrine resistance, our in vivo evidence confirms that MDK inhibition (iMDK) significantly sensitizes tumors to the androgen receptor inhibitor enzalutamide. This synergistic effect suggests that iMDK could serve as a potent adjunct to overcome or bypass resistance in castration‐resistant prostate cancer (CRPC). Critically, MDK inhibition plays a pivotal role in remodeling the tumor immune microenvironment [44, 45]. While PCa is typically characterized as an immunologically “cold” tumor—refractory to immune checkpoint inhibitors (ICIs) due to its pervasive immunosuppressive landscape—our study highlights that iMDK alleviates these inhibitory signals by impeding LAM differentiation [46, 47]. Given that LAMs are instrumental in establishing immune barriers and impairing T‐cell function [40, 48], blocking MDK may ′re‐prime″ the anti‐tumor immune cycle. This strategy not only restores sensitivity to androgen receptor‐targeted therapies in CRPC but also holds the potential to convert the immunosuppressive environment into an immune‐supportive one, thereby augmenting the efficacy of immunotherapies such as anti‐PD‐1/PD‐L1. Furthermore, the present study underscores the central role of cholesterol in driving LAM differentiation and PCa progression via SP1 palmitoylation. Targeting this metabolic‐epigenetic axis represents a promising therapeutic avenue to mitigate tumor‐driven immune suppression. Further investigations into the mechanisms of cholesterol‐induced SP1 palmitoylation and its broader implications in cancer biology will be critical for developing targeted therapies to combat PCa and potentially other cholesterol‐driven malignancies.

## 4 Methods

### 4.1 Cell Lines and Cell Culture

LNCaP, 22RV1, RM1, and HEK‐293T cells were purchased from the Cell Bank of the Chinese Academy of Sciences (Shanghai, China). RPMI‐1640 (Gibco, Shanghai, China) was used for LNCaP, 22RV1, RM1, BMDM, and T cell culture, and DMEM (Gibco, Shanghai, China) was used for HEK‐293T cell culture according to the manufacturer's instructions in a humidified atmosphere of 5% CO_2_ at 37°C (BB150, Thermo Scientific, Shanghai, China), as described in a previous study.

### 4.2 Isolation and Culture of BMDMs and Spleen‐Derived T Cells

Bone marrow‐derived macrophages (BMDMs) and spleen‐derived T cells were isolated from C57BL/6 mice. Femurs and tibias were harvested aseptically and flushed with PBS, and bone marrow cells were collected. The cells were cultured in complete medium supplemented with 25 ng/mL M‐CSF for 7 days. Adherent cells were identified as BMDMs. For spleen‐derived T cells, the spleens were processed into single‐cell suspensions; RBCs were lysed, and T cells were isolated using magnetic beads or nylon wool. Purified T cells were resuspended at 1 × 10^6^ cells/mL in complete medium, stimulated with anti‐CD3/CD28, and then cultured at 37°C with 5% CO_2_.

### 4.3 RNA Isolation and qPCR

Total RNA was isolated with RNA‐Quick Purification Kits (ES‐RN001, Yishan, Shanghai, China) and a Tissue RNA Purification Kit Plus (ES‐RN002plus, Yishan, Shanghai, China). HiScript IV All‐in‐One Ultra RT SuperMix (R433‐01, Vazyme, Jiangsu, China) was applied to synthesize cDNA according to the manufacturer's instructions. The ChamQ Universal SYBR qPCR Master Mix (Vazyme) was used for quantitative real‐time PCR, which was conducted using an ABI QuantStudio Sequence Detection System (Applied Biosystems, Foster City, CA, USA). The primers used for qPCR are provided in the Supporting Information.

### 4.4 Cholesterol Measurement

Enzymatic assay kits (E1015, APPLYGEN, Beijing, China) were used to measure cholesterol levels in tissues and cell samples. The cells or tissues were lysed, and the lysates were centrifuged to obtain a clear supernatant. The supernatant was mixed with a working solution containing cholesterol esterase and oxidase, followed by incubation for 20 min at 37°C. A microplate reader (Multiskan MK3, Thermo Scientific, Shanghai, China) was used to measure the OD 550 nm. The cholesterol content was then normalized to the protein concentration per milligram or the number of cells. In addition, cholesterol was labeled with a filipin complex (HY‐N6716, MCE, Shanghai, China) and then detected by flow cytometry [49].

### 4.5 Immunohistochemical Staining Analysis

The SQLE and MDK protein levels in PCa tissues were detected with SQLE antibodies (12544‐1‐AP; Proteintech, Wuhan, China) and MDK antibodies (11009‐1‐AP; Proteintech, Wuhan, China), respectively, as described previously [50, 51]. A Nikon Eclipse 80i system (Nikon, Tokyo, Japan) was used to analyze the images. Three researchers independently quantified protein expression via immunohistochemistry. All human tissues were obtained from patients treated with radical prostatectomy, which was approved and supervised by the Ethics Committees of Sun Yat‐Sen Memorial Hospital (SYSEC‐KY‐KS‐2020‐201).

### 4.6 Western Blot Analysis

Protein was extracted from the cells and detected by Western blot analysis as previously described [52]. The following antibodies were purchased from Proteintech (China): SQLE (12544‐1‐AP), CD86 (13395‐1‐AP), CD206 (18704‐1‐AP), GAPDH (10494‐1‐AP), SP1 (21962‐1‐AP), MDK (11009‐1‐AP), H3 (29200‐1‐AP), TREM2 (83438‐6‐RR), CD36 (66395‐1‐Ig), goat anti‐rabbit IgG (1:10,000), and anti‐mouse IgG (1:10 000). The blots were visualized with an enhanced chemiluminescence (ECL) detection system (PK10003, Proteintech).

### 4.7 Cell Proliferation Assay

CCK8 assays were used to determine the proliferation of LNCaP, 22RV1 and RM1 cells according to the manufacturer's protocol. The cells were cultured in 96‐well plates and treated with cholesterol. To each well, 10 µL of CCK8 was added, and the mixture was incubated for 2 h in the dark before detection.

### 4.8 Immunofluorescence (IF) Staining

The cells were fixed with 4% paraformaldehyde and then treated with 0.5% Triton X‐100 for permeabilization. After incubation with specific primary antibodies overnight at 4°C, the cells were washed and then incubated with secondary antibodies for 1 h at room‐temperature. DAPI (Beyotime, Shanghai, China) was used for nuclear staining [53]. A confocal microscope (Zeiss, Munich, Germany) was used to capture the images.

### 4.9 Nuclear and Cytoplasmic Protein Extraction

The Nuclear and Cytoplasmic Protein Extraction Kit (P0027, Beyotime Biotechnology, Shanghai, China) was used according to the manufacturer's instructions. Briefly, the cells were mixed with cytoplasmic protein extraction reagent A and vortexed vigorously at high speed, followed by incubation on ice for 30 min. The samples were subsequently mixed with cytoplasmic protein extraction reagent B and vortexed again at high speed. The cytoplasmic proteins in the supernatant were collected after centrifugation. The remaining precipitate was mixed with nuclear protein extraction reagent and vortexed vigorously at high speed, followed by incubation on ice. During this period, the sample was vortexed every 12 min, and this process was continued for a total of 30 min. After incubation in an ice bath, the supernatant containing the nuclear proteins was obtained by centrifugation.

### 4.10 Chromatin Immunoprecipitation (ChIP) Assay

The ChIP assay kits (P2078, Beyotime Biotechnology, Shanghai, China) were used according to the manufacturer's instructions. The cells were fixed with 1% formaldehyde for 10 min, and the crosslinking reaction was halted with glycine treatment for 5 min. The cells were then lysed using SDS lysis buffer, and the chromatin was sonicated to produce fragments ranging from 200 to –1000 bp. Nonspecific binding was minimized by preclearing with protein A+G agarose combined with salmon sperm DNA. A 2% sample of chromatin was set aside as an input control, while the remaining sample was incubated overnight at 4°C with IgG and SP1 antibodies. The immune complexes were captured and washed with buffers, and the crosslinks were reversed with NaCl. After DNA was extracted and purified with RNase A and proteinase K, it was quantified by qPCR.

### 4.11 Dual‐Luciferase Reporter Assay

The following plasmids were transfected into cells according to experimental requirements: pcDNA3.4‐SP1, pRL‐TK‐R‐Luc, pGL3‐Basic, and pGL3‐Basic with the WT MDK promoter or a mutant MDK promoter (IGE Biotechnology, Guangzhou, China). Regarding the MDK promoter mutants, the G/C base was mutated to an A base. For Homo sapiens, the following mutations were generated: Mut1, GGGGCGGGG was mutated into AAAACAAAA at positions 1955–1963; Mut 2, GGGGCGGGA was mutated into AAAACAAAA at positions 1704–1712; and Mut 3, GGGGAGGAG was mutated into AAAAAAAAA at positions 917–925. For Mus musculus, the following mutations were generated: Mut1, CCCCTCCCCC was mutated into AAAATAAAAA at positions 1458–1467; Mut 2, CCCCCCCCCC was mutated into AAAAAAAAAA at positions 1479–1488; and Mut 3, CCCCCCCCCC was mutated into AAAAAAAAAA at positions 1485–1494. At 40 h after transfection, the cells were lysed and detected with a Dual Luciferase Reporter Gene Assay Kit (Yeasen Biotechnology Shanghai, China) according to the manufacturer's instructions.

### 4.12 Acyl‐Biotin Exchange (ABE) Assay

A previously reported method, with slight modifications, was used for the ABE assay [54]. Initially, total protein lysates were incubated with 25 mm N‐ethylmaleimide (NEM; Aladdin, Shanghai, China) overnight at 4°C. This step serves to completely block free thiol groups on cysteine residues, preventing non‐specific background labeling in subsequent steps. Following the blockade, chloroform‐methanol precipitation was carried out to remove residual buffer and excess unreacted NEM, which could interfere with downstream reactions. The protein pellets were then resuspended and treated with 1 m hydroxylamine (Aladdin, Shanghai, China) at pH 7.4 for 1 h at 25°C. The purpose of this treatment is to specifically cleave the thioester bonds of palmitoylated cysteines, thereby re‐exposing the thiol groups that were previously modified by palmitic acid. The newly exposed thiols were then selectively labeled using HPDP‐biotin (Aladdin, Shanghai, China) at room‐temperature for 1 h. To ensure specificity, unreacted HPDP‐biotin was removed by a second round of chloroform‐methanol precipitation (optional but recommended for clarity). The biotinylated proteins—representing the palmitoylated fraction—were then affinity‐purified/enriched using streptavidin agarose beads (Beyotime, Shanghai, China). Finally, the beads were washed thoroughly to eliminate nonspecifically bound proteins. The enriched proteins were eluted by boiling at 95°C for 10 min in SDS sample buffer and were subsequently analyzed via SDS‐PAGE and immunoblotting.

### 4.13 Animal Studies

All animal experiments were conducted at the Experimental Animal Center of Sun Yat‐sen Memorial Hospital, approved and supervised by the Animal Ethics Committee of Sun Yat‐sen Memorial Hospital (AP20230225). Male C57BL/6 mice (6–8 weeks old) were housed under specific pathogen‐free (SPF) conditions with free access to food and water. To investigate the impact of cholesterol on tumor progression, mice were fed either a normal diet (ND) or a high‐cholesterol diet (HCD, 3% cholesterol) for 12 weeks prior to tumor inoculation, with respective diets maintained throughout the experiment. For the tumor xenograft model, RM1 cells (1 × 10^6^ cells per mouse, suspended in 100 µL PBS) were subcutaneously implanted into the flank of the mice. To evaluate combinatorial therapeutic efficacy, tumor‐bearing mice were randomized (*n* = 5) and treated via intraperitoneal injection with Enzalutamide (10 mg/kg daily; HY‐70002, MCE) either alone or in combination with the following agents: Simvastatin (20 mg/kg daily; HY‐17502, MCE), Terbinafine (30 mg/kg daily; HY‐17395, MCE), or the MDK inhibitor iMDK (9 mg/kg daily; HY‐110171, MCE). Tumor volume was monitored and calculated as *V* = length × width^2^ × 0.5.

Macrophages were depleted in vivo using clodronate liposomes (Yeasen Biotechnology, 40337ES10). Mice were administered 200 µL of clodronate liposomes (5 mg/mL) or control PBS liposomes via intraperitoneal injection 24 h prior to tumor cell inoculation, followed by maintenance doses of 100 µL every 4 days throughout the experiment. Depletion efficiency was verified by flow cytometry analysis of tumor‐infiltrating immune cells using APC Anti‐Mouse/Human CD11b Antibody (Elabscience, E‐AB‐F1081E) and PE Anti‐Mouse F4/80 Antibody (Elabscience, E‐AB‐F0995D).

### 4.14 Statistical Analysis

The quantitative results are expressed as the mean ± standard deviations (SD) (*n* = 3), except for in vivo studies (*n* = 5) and were analyzed using GraphPad Prism 9.0 software (GraphPad, La Jolla, CA, USA). Statistical differences between two groups were determined by Student's *t*‐test, whereas comparisons across multiple groups were assessed via one‐way ANOVA with Dunnett's post hoc test for multiple comparisons. A *P* value less than 0.05 was set as the threshold for statistical significance.

## Author Contributions

S.R.P., K.L., and H.H. conceived and designed the study. S.R.P., Z.L., S.M.P., and R.Z. conducted the experiments and performed data analysis and interpretation. B.L., B.C., Y.L., Y.O., and W.Z. provided supervision. The original draft of the manuscript was written by S.R.P., Z.L., S.M.P., and R.Z. Manuscript review and editing were carried out by S.R.P. and Z.L. Article revision and experimental supplementation were performed by S.R.P. and W.L.L. All authors read and approved the final manuscript.

## Conflicts of Interest

The authors declare no conflicts of interest.

## Supporting information




**Supporting File**: advs74081‐sup‐0001‐SuppMat.docx.

**Supporting File**: advs74081‐sup‐0002‐TableS1‐S2.xlsx.

## Data Availability

The data that support the findings of this study are available from the corresponding author upon reasonable request.
