# Supplementary material for: Cancer Cell‐Intrinsic Cholesterol Induces Lipid‐Associated Macrophage Differentiation via SP1 Palmitoylation to Promote Prostate Cancer Progression
Source: Adv Sci (Weinh). 2026 Jan 28;13(19):e08588. doi: 10.1002/advs.202508588 (PMC13045348; doi:10.1002/advs.202508588)
Supplement: Supplementary file 1 — Supporting File: advs74081‐sup‐0001‐SuppMat.docx. [file ADVS-13-e08588-s001.docx]

**
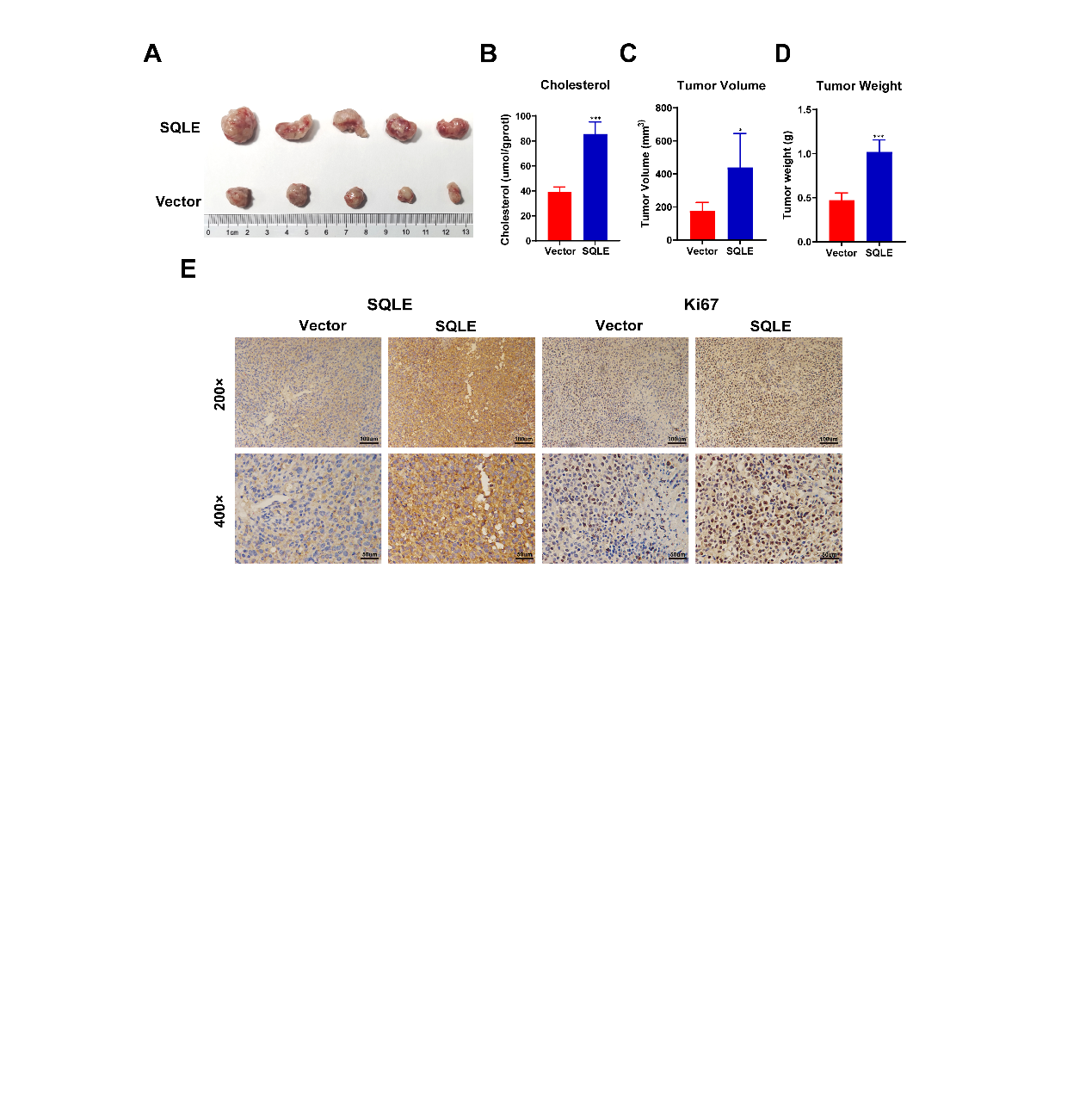
****Figure S1 Overexpression of SQLE promotes prostate cancer progression *in vivo.***

**A** Representative image of xenograft tumors (*n* = 5 per group). **B-D** Quantitative analysis of cholesterol content, size, and weight of tumors. **E** Representative immunohistochemical images of SQLE and Ki67 expression in tumor tissues. Data are presented as mean ± SD (*n* = 5). Statistical differences between two groups were determined by Student’s t test. **P* < 0 .05, ***P* < 0 .01, and ****P* < 0.001, Scale bar: 100 µm for 200X, 50 µm for 400X.

**
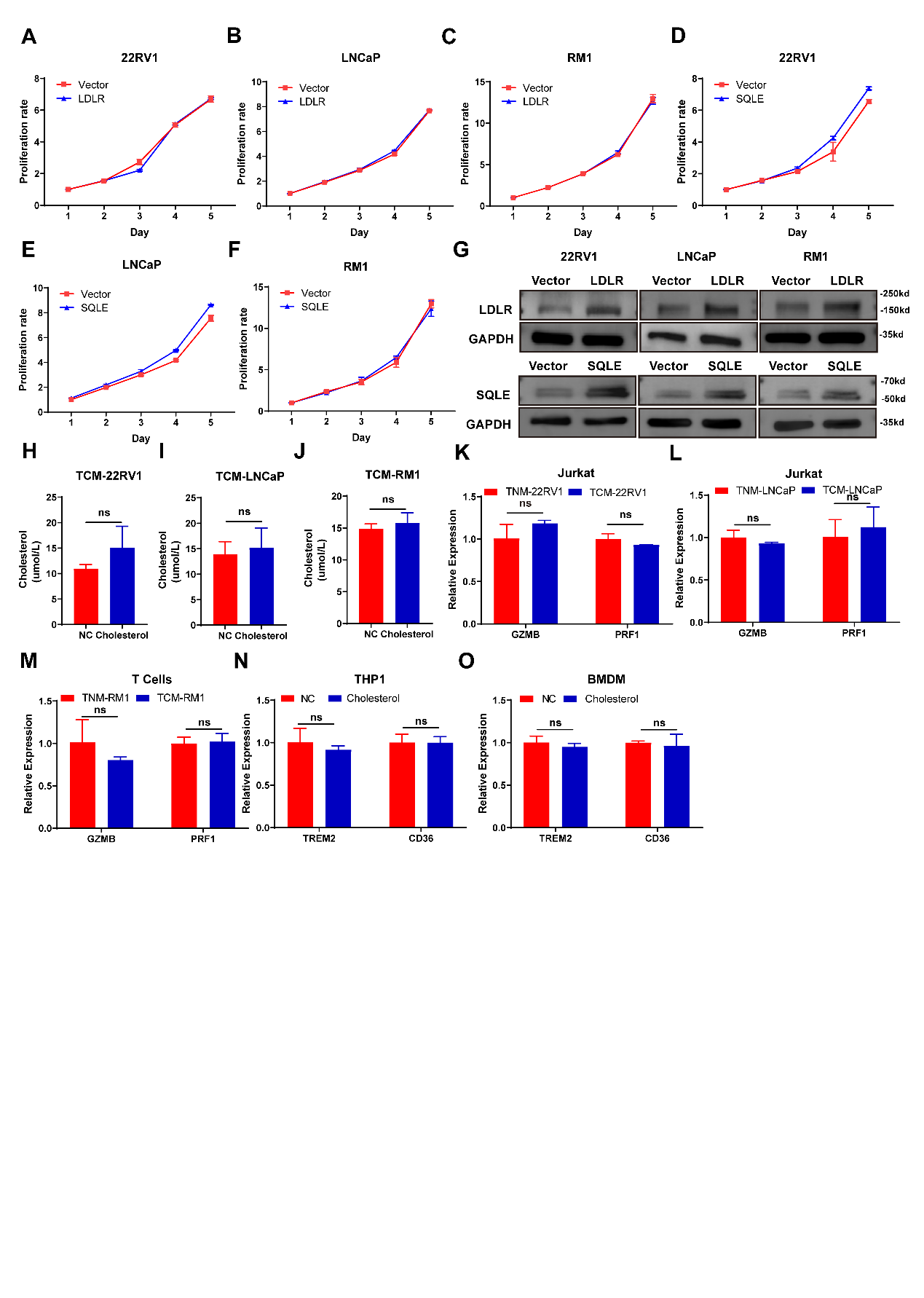
**

**Figure S2 The tumor-conditioned medium of cholesterol pretreated tumor cells does not promote the cytotoxic activity of T cells.**

**A-G** LDLR and SQLE overexpression did not significantly promote the growth of PCa cells *in vitro.* **H-J** Quantification of cholesterol content in the culture medium of tumor cells treated with or without cholesterol. **K-M** Quantitative PCR analysis of expression changes in GZMB and PRF1 in T cells following treatment with different tumor-conditioned medium. **N-O** Quantitative PCR analysis demonstrated that cholesterol did not increase the mRNA levels of TREM2 and CD36 in macrophages. Data are presented as mean ± SD (*n* = 3). Statistical differences between two groups were determined by Student’s t test. **P* < 0.05, ***P* < 0.01, ****P* < 0.001, and ns for non-significant.


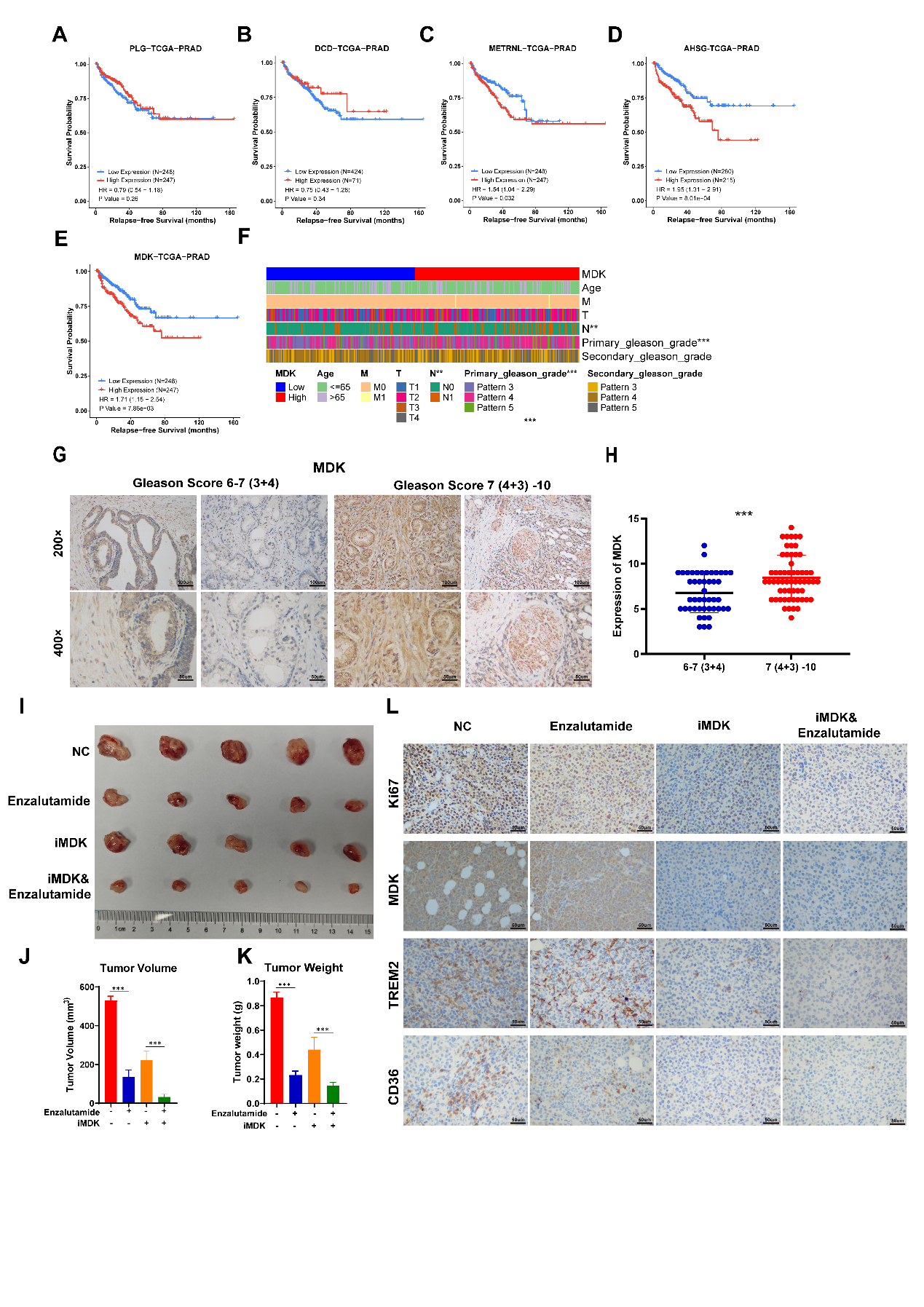


**Figure S3 Prognostic significance of different cytokines and the therapeutic efficacy of MDK inhibition in PCa.**

**A-E** Kaplan-Meier analysis of the association between expression levels of PLG (**A**), DCD (**B**), METRNL (**C**), AHSG (**D**), and MDK (**E**) and relapse-free survival in prostate cancer patients. **F** TCGA data analysis suggested that MDK expression was associated with high lymph node metastasis and Gleason score. **G-H** Representative IHC images (G) and quantification (H) of MDK protein expression in the Sun Yat-sen Memorial Hospital cohort (*n* = 105), stratified by Gleason scores. **I-L** MDK inhibitor (iMDK) significantly inhibited LAM differentiation and attenuated prostate tumor growth, enhancing the sensitivity of tumors to the androgen receptor inhibitor enzalutamide *in vivo* (*n* = 5 per group). Scale bar: 50 µm. Data are presented as mean ± SD. Statistical differences between two groups were determined by Student’s t test (H), whereas comparisons across multiple groups were assessed via one-way ANOVA with Dunnett’s post hoc test for multiple comparisons (J, K). **P* < 0.05, ***P* < 0.01, and ****P* < 0.001.

**
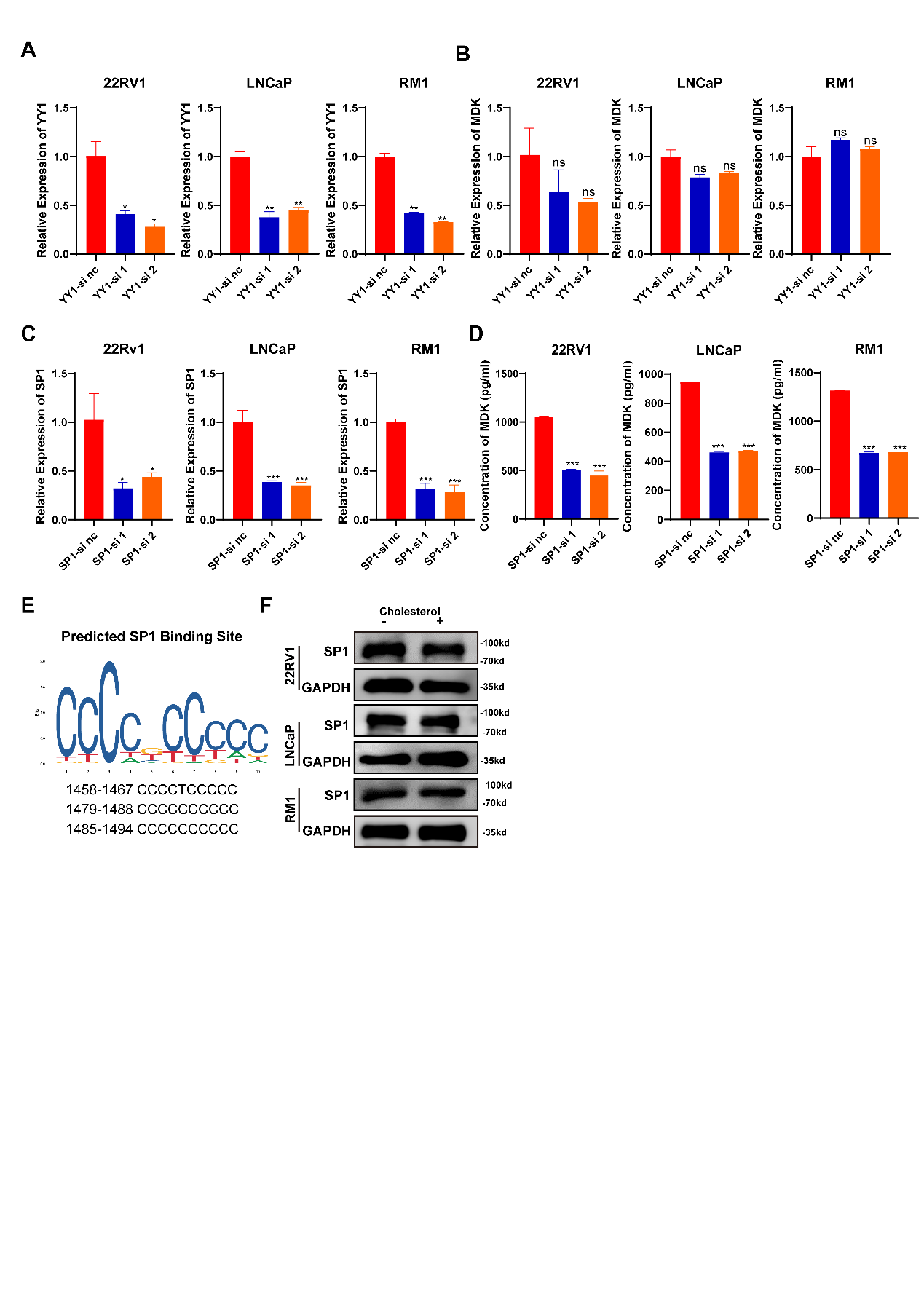
**

**Figure S4 SP1, but not YY1, mediates cholesterol-induced MDK expression.**

**A** qPCR confirmation of YY1 knockdown. **B** qPCR analysis of changes in MDK expression following YY1 knockdown. **C** qPCR confirmation of SP1 knockdown. **D** ELISA showed that silencing SP1 attenuated MDK secretion by prostate cancer cells. **E** Prediction of potential SP1 binding sites within the MDK promoter region in Mus musculus, as identified through the JASPAR database. **F** Western blot analysis confirmed that cholesterol does not affect total SP1 protein levels in tumor cells. Data are presented as mean ± SD (*n* = 3). Statistical differences between two groups were determined by Student’s t test (siNC as control). **P* < 0.05, ***P* < 0.01, ****P*< 0.001, and ns for non-significant.


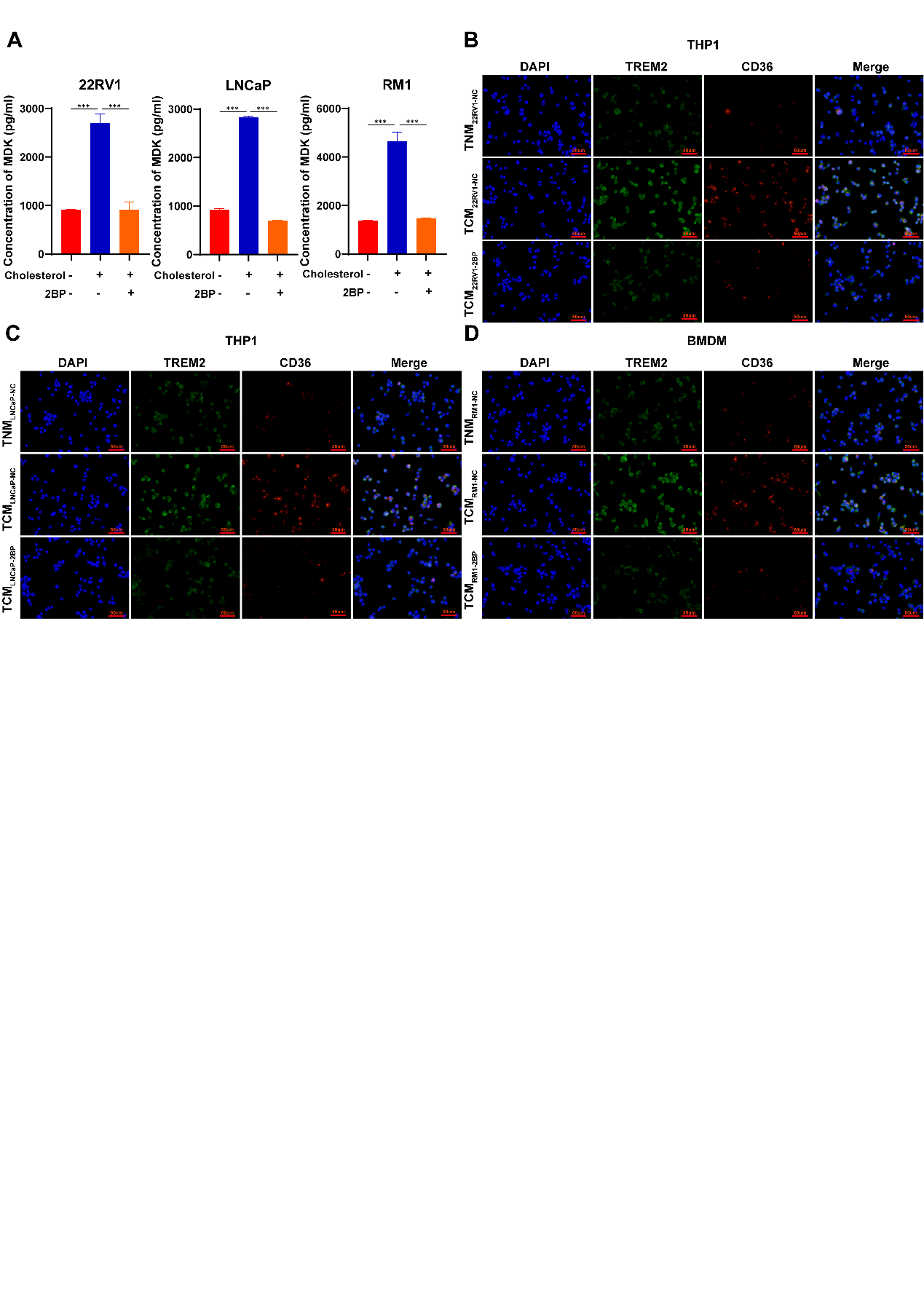


**Figure S5 2BP treatment in prostate cancer cells reverses the differentiation of lipid-associated macrophages.**

**A** Tumor cells were co-treated with or without cholesterol and 2-bromopalmitate (2BP), and the conditioned medium was collected for ELISA of MDK. **B-C** Tumor cells were co-treated with or without cholesterol and 2-bromopalmitate (2BP), and the conditioned medium was collected to stimulate macrophage differentiation. Immunofluorescence staining was performed to detect TREM2 and CD36 expression in macrophages. Scale bar, 50 µm. Data are presented as mean ± SD (*n* = 3). Statistical differences between multiple groups were assessed via one-way ANOVA with Dunnett’s post hoc test for multiple comparisons. **P* < 0.05, ***P* < 0.01, and ****P* < 0.001.


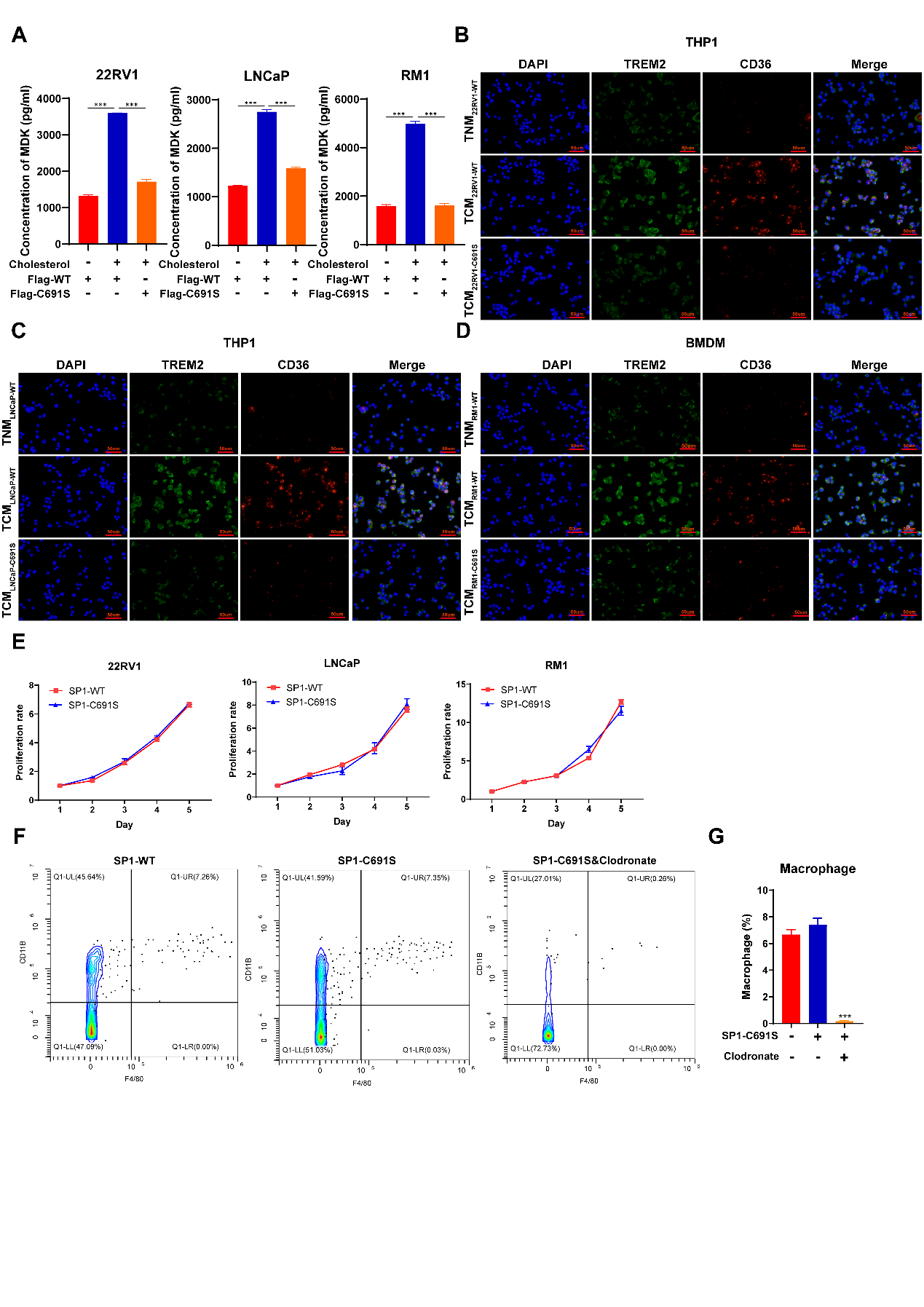


**Figure S6 Cys691S mutation in PCa cells reverses the differentiation of lipid-associated macrophages induced by tumor-conditioned medium.**

**A** Conditioned medium from PCa cells overexpressing either wild-type (WT) SP1 or Cys691S SP1 was collected for ELISA of MDK. **B-D** Conditioned medium from PCa cells overexpressing either wild-type (WT) SP1 or Cys691S SP1 was collected to stimulate macrophage differentiation. Immunofluorescence staining was performed to assess TREM2 and CD36 expression in macrophages. **E** Cys691S SP1 did not significantly inhibit the growth of PCa cells *in vitro.* **F** Flow cytometry confirms clearance of macrophages *in vivo*. Scale bar, 50 µm. Data are presented as mean ± SD (*n* = 3), except for *in vivo* studies (n = 5). Statistical differences between multiple groups were assessed via one-way ANOVA with Dunnett’s post hoc test for multiple comparisons. **P* < 0.05, ***P* < 0.01, and ****P* < 0.001.


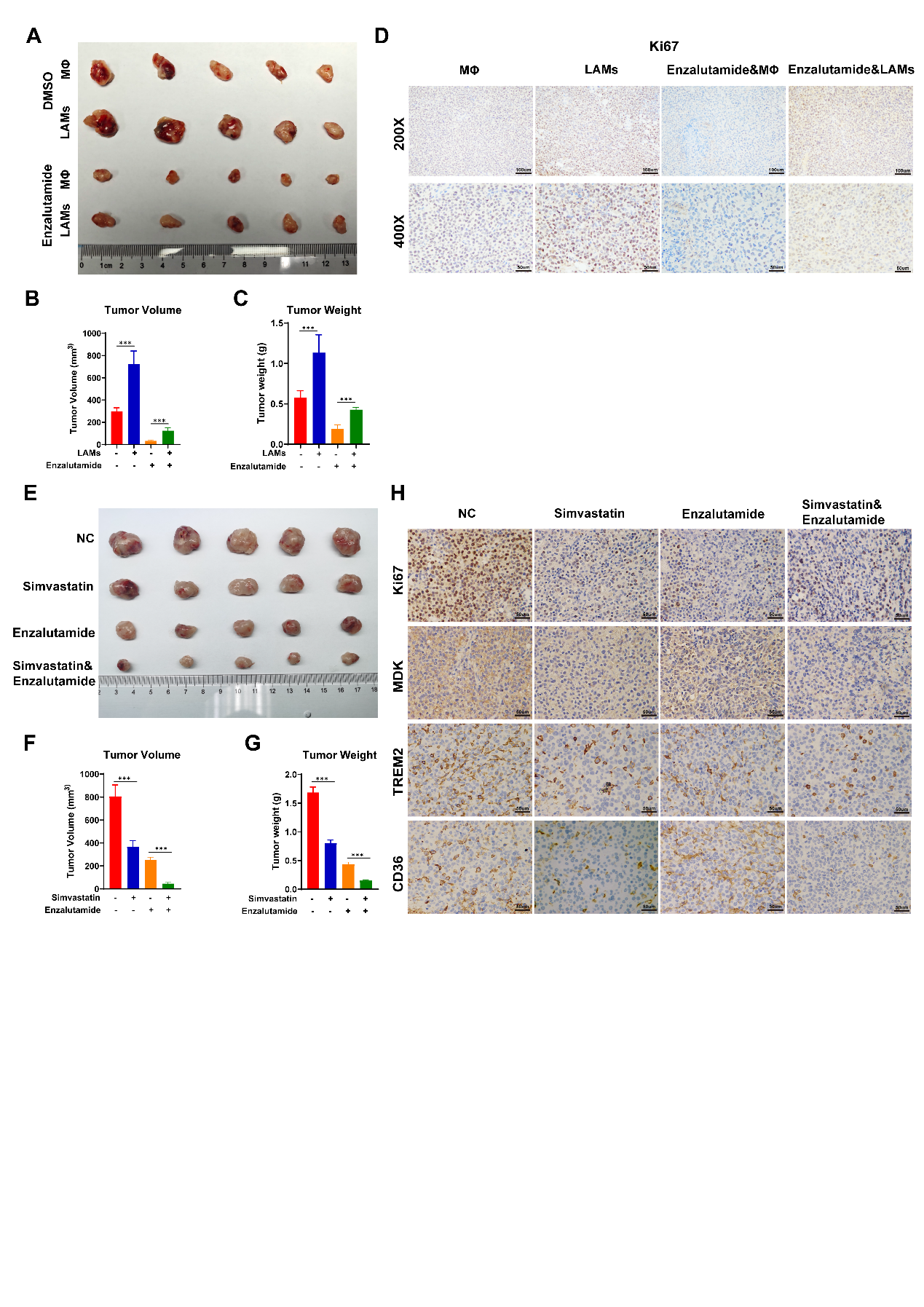


**Figure S7 Targeting cholesterol metabolism and LAMs enhances the effectiveness of enzalutamide in PCa.**

**A-D** LAMs promoted tumor growth and reduced tumor sensitivity to enzalutamide *in vivo* (*n* = 5 per group). **E-H** Targeting cholesterol metabolism in cancer cells using Terbinafine enhanced the efficacy of enzalutamide treatment *in vivo* (*n* = 5 per group). Scale bar, 50 µm. Data are presented as mean ± SD (*n* = 5). Statistical differences between multiple groups were assessed via one-way ANOVA with Dunnett’s post hoc test for multiple comparisons. **P* < 0.05, ***P* < 0.01, and ****P*< 0.001.
